# Supplementary material for: Functional analysis of TTN uORFs reveals context-dependent translational regulation
Source: Mol Ther Nucleic Acids. 2026 Aug 28;37(4):103077. doi: 10.1016/j.omtn.2026.103077 (PMC13586513; doi:10.1016/j.omtn.2026.103077)
Supplement: Supplementary file 1 [file mmc1.pdf]

OMTN, Volume *37*

Supplemental information

Functional analysis of *TTN* uORFs reveals context-dependent translational regulation

Rosemary B. Kirk, Alexander J. Sparrow, Marta Moya-Jódar, Thomas C. Roberts, Christopher N. Toepfer, and Hugh Watkins

Supplemental material

Table S1: Engineered hiPSC lines do not show predicted off-target mutations.

The guide RNA used for engineering of hiPSC lines was assessed for predicted off-target mutations using CRISPOR. PCR primers were designed for the top five off-target regions and genotyping performed on all subclones.

Loci (hg19)	Off target score	Mismatch	Genomic location	Result			
				WT	WT-ΔuORF	*TTN*+/-	*TTN*+/--ΔuORF
Chr2:215157979-215158001	0.535	AGTGGAACACCAA TTCATGT(TGG)	Intergenic	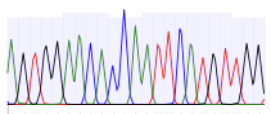	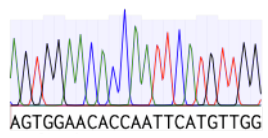	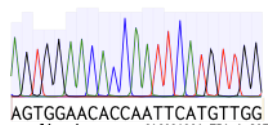	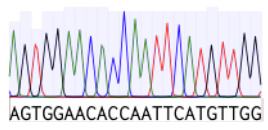
Chr5:17271161-17271183	0.533	ACAGGCTTACCG ATTAATAT(TGG)	Intergenic	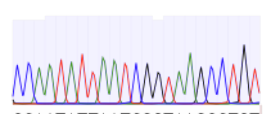	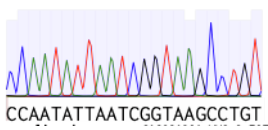	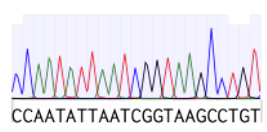	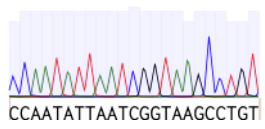
Chr3:172667593-172667615	0.528	AGAGACTCATCA ATTCATGA(GGG)	Intron	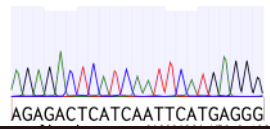	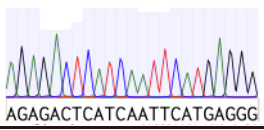	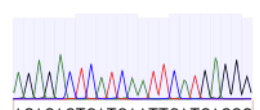	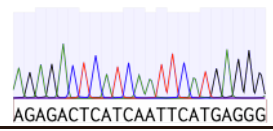
Chr11:20088531-20088553	0.369	GCAGGCTCCCCA ATTCATGT(GGG)	Intron	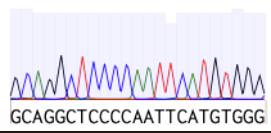	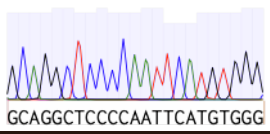	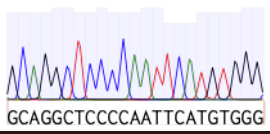	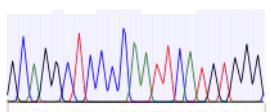
Chr18:66515999-66516021	0.320	AGAGGATCAAAC ATTCATGT(AGG)	Intergenic	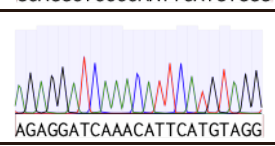	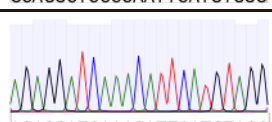	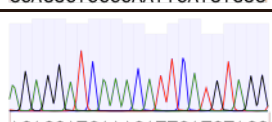	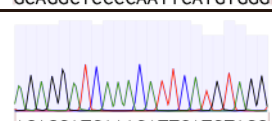

Table S2: Primers used for genotyping and RT-qPCR.

Target gene	Application	Sequence (5'-3')	Notes
Renilla luciferase	RT-qPCR	F: GTAACGCTGCCTCCAGCTAC<br>R: CCAAGCGGTGAGGTACTTGT	Quantification of mRNA levels of *Renilla* luciferase from dual reporter assays (under 5'-UTR control).
Firefly luciferase	RT-qPCR	F: ACTCTAAGACCGACTACCAGG<br>R: GTAGACCCAGAGCTGTTCATG	Quantification of mRNA levels of Firefly luciferase from dual reporter assays (internal control).
TTN	Genotyping	F: TAGGTCCTCTCCCATCATGCTT<br>R: ACAGGCAAAGAGAGTCTAGAAAAGA	For PCR and sequencing of *TTN* uORF region to genotype hiPSC lines.
TTN	Genotyping	F: CTCCCCAATATTTTTCGTTCACTCT<br>F_int1: TGCCTATCAGTTTCAGAGACATT<br>F_int2: GATGAACAACTTTCTGTGTCTAACT<br>F_int3: GGGGCTGAAGGTATGCGTCT<br>F_int4: GGTCAGCACTGGCAAGAAATAG<br>R_int3: CGCCTCGGGTGTAGGGATT<br>R_int2: CTTCTCTGAGCCTCAACACAGT<br>R_int1: CTGTCCCCGAAATACAAGTGGA<br>R: CACCTCCTATCCACTTCCCATT	For PCR and sequencing of *TTN* uORF region inclusive of flanking SNVs. F and R used for PCR amplification and sequencing, internal (“int”) primers used for further sequencing to confirm no sequence drop-off within the product.
Off-target 1	Genotyping	F: CATGCCTTACCTTGAGCCGTA<br>R: GGATTACCAGACCAGCTCACC	
Off-target 2	Genotyping	F: TGTGTGGAACCTGTGTGCTAA<br>R: AAGCTAATTCTGGAGCTGTCCTT	
Off-target 3	Genotyping	F: TCTTCAGGGATTTTAATGCTTGGC<br>R: GAGTTGAGGTAGGGTAGGCA	
Off-target 4	Genotyping	F: GTGAACTAATGGAGATGCTGACA<br>R: AATGTGGCCTTCATCTTCGTG	
Off-target 5	Genotyping	F: AATGCATTTGAAAGTCTGGTGTGC<br>R: ACAATGCCTGTGTCTCTGAAAGTA	

Table S3: TaqMan probes used for RT-qPCR.

Target gene	Probe ID	Supplier	Reporter dye
TTN	Hs00399225_m1	Thermo Fisher Scientific	FAM
ACTN2	Hs00153809_m1	Thermo Fisher Scientific	VIC

Table S4: Antibodies used for immunofluorescence.

Target protein	Host species	Supplier	Catalogue no.	Dilution	Application
Nanog (D73G4)	Rabbit	Cell Signaling Technology	#99399	1:100	IF
Oct4 (C30A3)	Rabbit	Cell Signaling Technology	#94310	1:100	IF
Sox2 (D6D9)	Rabbit	Cell Signaling Technology	#34516	1:100	IF
Titin (M-line)	Rabbit	Myomedix	#TTN-9	1:100	IF
α-actinin (EA53)	Mouse	Sigma-Aldrich	#A7732	1:400	IF
Secondary (anti-rabbit Alexa Fluo 488)	Goat	Invitrogen	#A11008	1:200	IF
Secondary (anti-mouse Alexa Fluo 568)	Goat	Invitrogen	#A11019	1:200	IF

Table S5: guide RNA and HDR sequence for ΔuORF CRISPR-Cas9 gene-editing.

Component	Sequence
Guide	AGAGGCTCACCGATTCATGT
HDR	GCTCACCTGATTTCTCAAGAGTGCCTAAAAAGGGTGGGACTAAGCCCAAGGTTGCTTCTGAAACGACG TCCTATGGAGAGTTGGTTTTTCTGACCTTCTCCGACTTGAATCGGTGAGCCTCTAATCCTAAAAACAAAA CTACACAGTCAAGACTGTGTAGTTTTGCTTTTCTATGCAATCCCTACACCCGAGGCGAGGCT

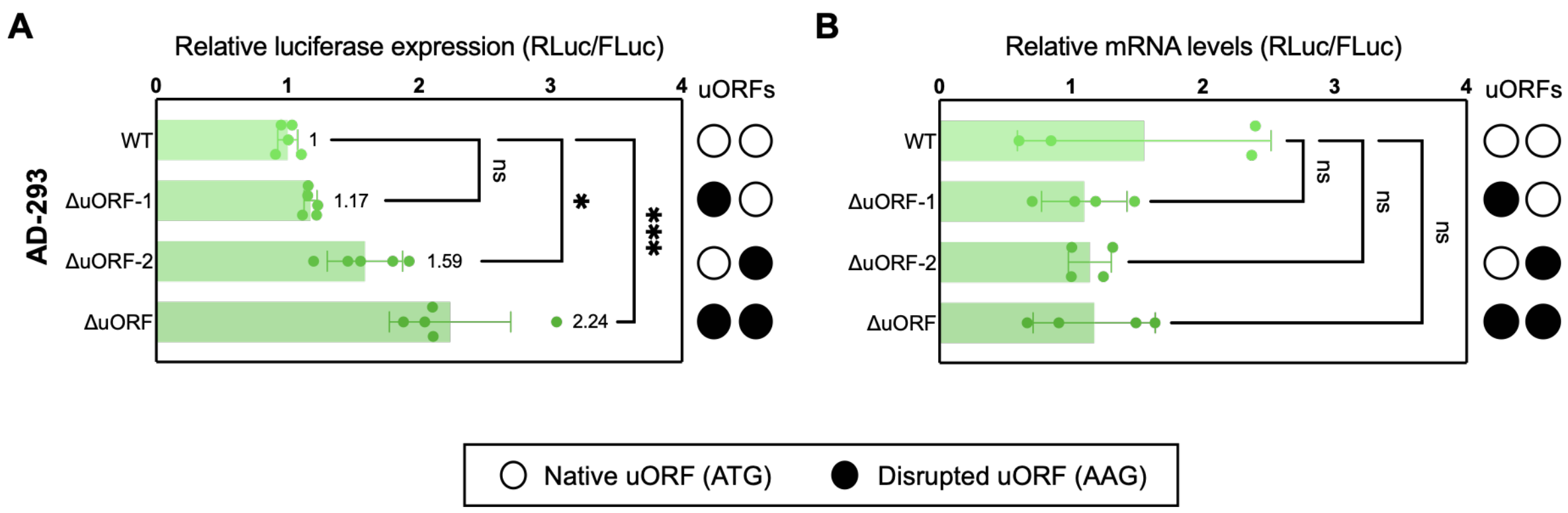

Figure S1: Individual disruption of *TTN* uORFs in a dual luciferase reporter assay in AD-293 cells.

(A) AD-293 cells were transfected with *TTN* 5' UTR dual luciferase reporter constructs containing the *TTN* 5' UTR in wild-type form (WT), with uORF-1 disrupted (ΔuORF-1), uORF-2 disrupted (ΔuORF-2), or both uORFs disrupted (ΔuORF). Luciferase activity was measured 24 hours post-transfection.

(B) AD-293 cells were transfected as in (A) and harvested 24 hours post-transfection for analysis of *Renilla* and firefly luciferase mRNA levels by RT-qPCR.

Bars represent mean (SD); n = 5 biological replicates each containing 4 technical replicates for (A) and n = 4 biological replicates each containing 2 technical replicates for (B). Values were normalised such that the mean of the WT control group was returned to a value of 1. Statistical significance was determined by performing a Kruskal-Wallis test with Dunnett's multiple comparisons test. *$p < 0.05$, ***$p < 0.001$; ns, not significant.

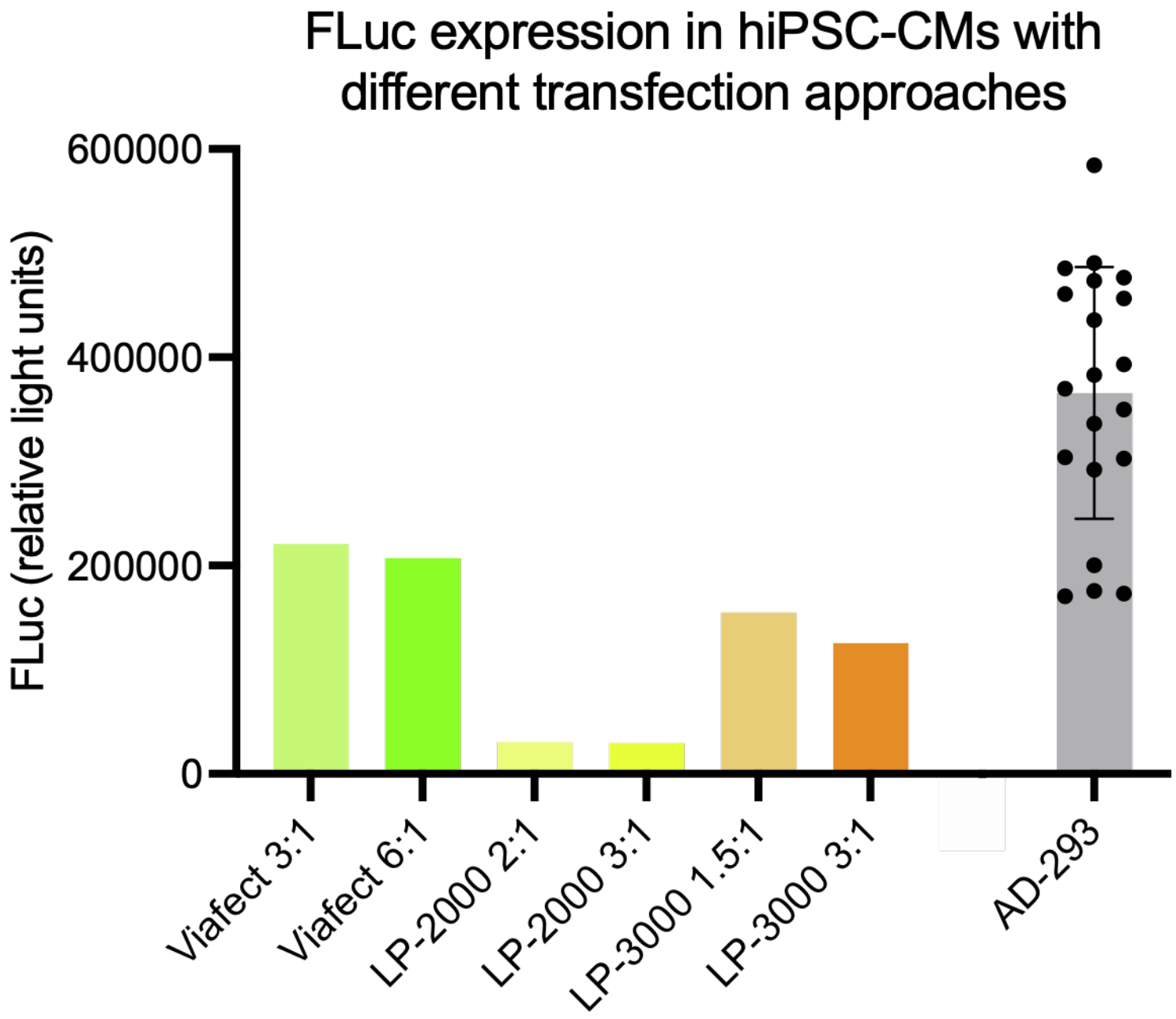

Figure S2: Optimisation of transfection conditions for dual luciferase reporter assays in hiPSC-derived cardiomyocytes.

Raw firefly luciferase activity measured 24 hours after transfection of hiPSC-CMs with the dual luciferase reporter plasmid using ViaFect, Lipofectamine 2000 (LP-2000), or Lipofectamine 3000 (LP-3000) at two reagent:DNA ratios (n = 1 per condition). Raw firefly luciferase activity from AD-293 cells transfected with the same reporter construct under identical assay conditions is shown for comparison (n = 20; 4 technical replicates from 5 independent experiments).

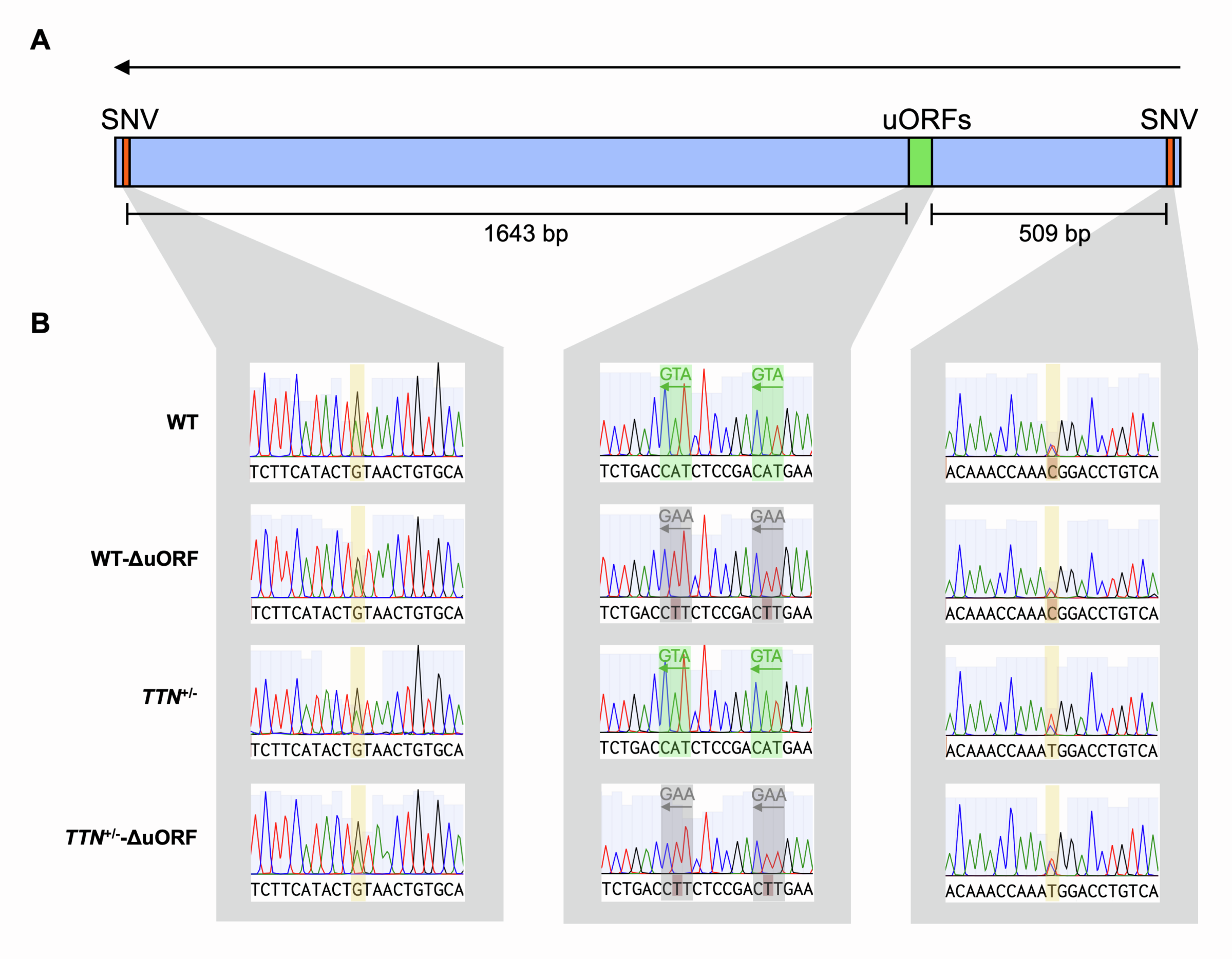

Figure S3: Engineered hiPSC lines show successful introduction of homozygous uORF disruption without evidence of large indels.

To exclude the possibility that apparently homozygous sequencing traces reflected monoallelic editing with a large indel in the second allele, the closest upstream and downstream heterozygous SNVs (**A**) were sequenced to confirm the presence of both alleles. Representative sequencing traces of the SNVs and the edit site are shown for all generated cell lines (**B**). Heterozygous SNVs are highlighted in yellow, uORF start codons in green, and disrupted uORF start codons in grey. As *TTN* is encoded on the reverse strand, the complementary sequence of the uORF start codons is annotated.

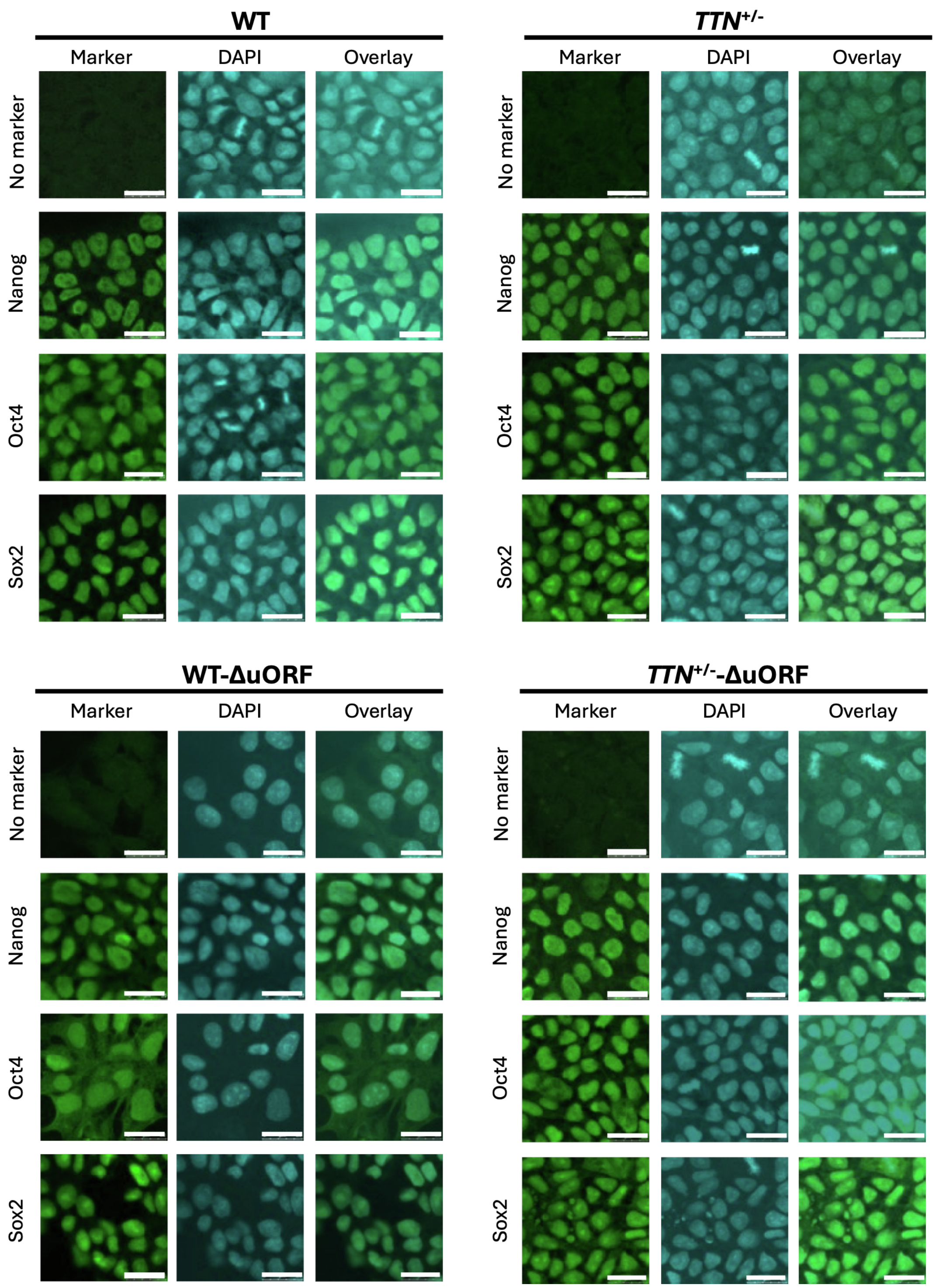

Figure S4: Engineered hiPSC lines express key pluripotency markers.

hiPSCs from all four generated cell lines (WT, WT-ΔuORF, *TTN*$^{+/-}$, *TTN*$^{+/-}$-ΔuORF) were fixed with 4% PFA/DPBS and incubated with rabbit IgG monoclonal antibodies targeting pluripotency markers Nanog, Oct4, and Sox2. All were incubated with secondary antibody and with DAPI as a nuclear marker, and imaged by confocal microscopy. Images show co-localisation of each pluripotency marker (green) with nuclear DAPI-stained DNA (blue). Images acquired with a 63× oil immersion objective with 3× magnification. Scale bar (white) 20 μm.

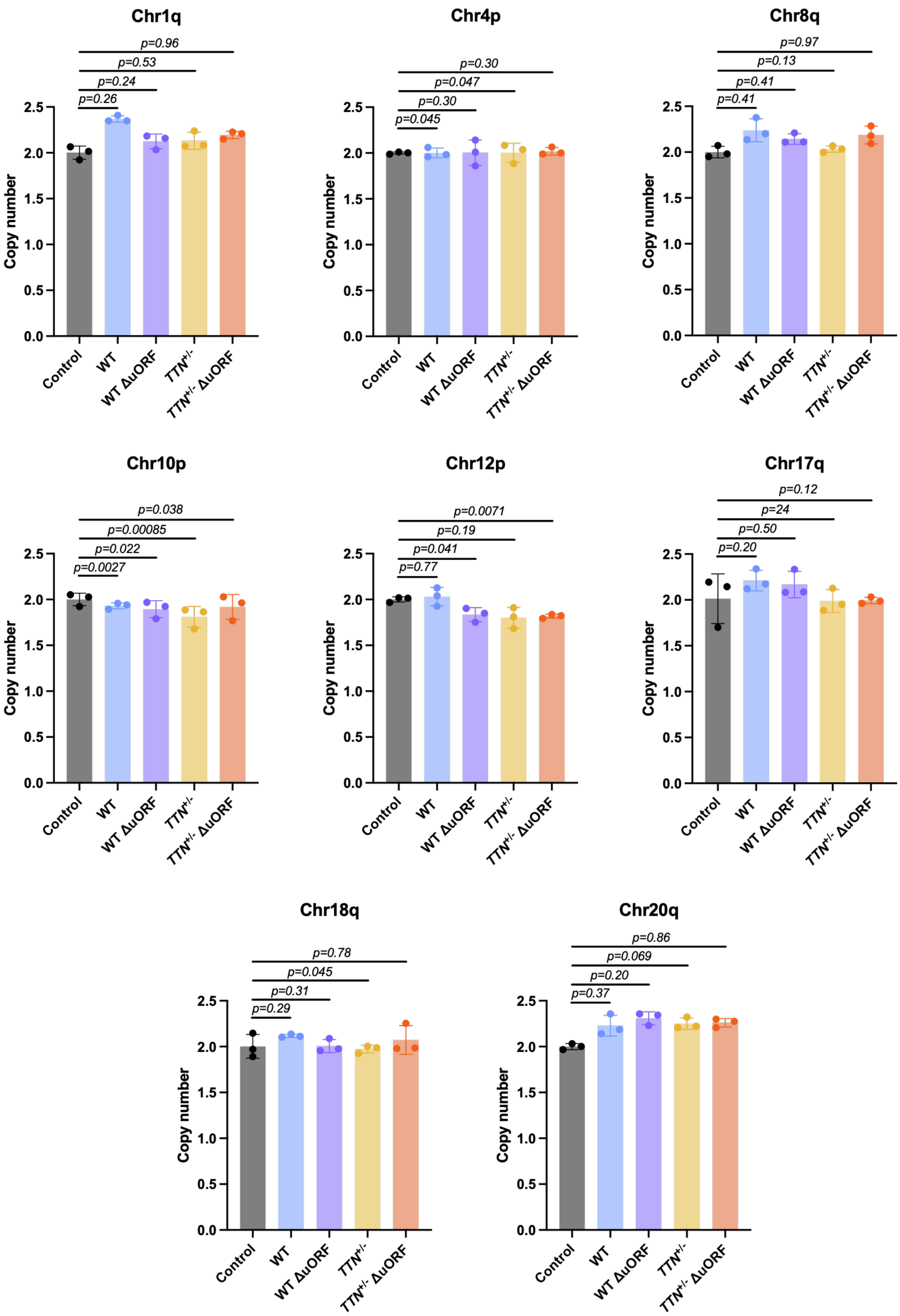

Figure S5: Engineered hiPSC lines do not have common hiPSC karyotype abnormalities.

Copy number analysis of genomic regions frequently affected in hiPSC culture was performed for each engineered hiPSC line (WT, WT-ΔuORF, *TTN*$^{+/-}$, *TTN*$^{+/-}$-ΔuORF) compared to a company-provided control using RT-qPCR. Bars represent mean (SD); n = 3 technical replicates. Data were analysed using the STEMCELL Technologies analysis platform, which applies a gradient-boosted machine learning algorithm to classify samples based on copy number and statistical significance (ANOVA). *p*-values are shown. No samples were classified as having putative or definitive chromosomal amplifications or deletions.

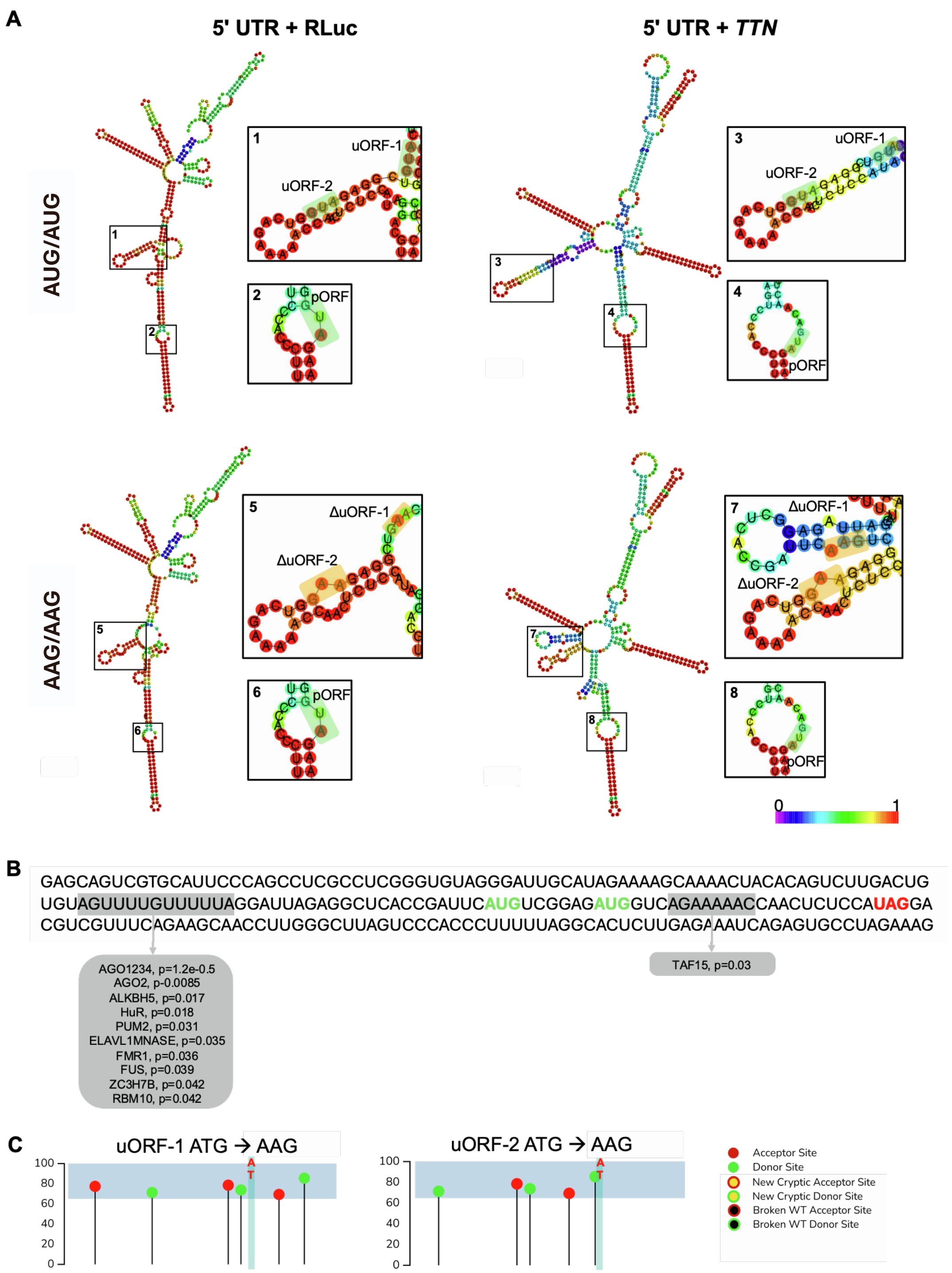

Figure S6: *In silico* analyses do not predict that altered sequence context or uORF disruption alter gene expression via alternative mechanisms.

(A) RNA secondary structure predictions generated using RNAfold for the *TTN* 5' UTR followed by either *Renilla* luciferase or *TTN* sequence (400 bp total), with wild-type uORF sequence (AUG/AUG) or uORF disruption (AAG/AAG). No major changes in predicted secondary structures were observed.

(B) Analysis of RNA-binding motifs within the *TTN* 5' UTR using BRIO (BEAM RNA Interaction mOtifs) shows several motifs in the 5' UTR, but none that would be disrupted by mutagenesis of the uORF start codons.

(C) Human Splicing Finder analysis predicting the effects of the nucleotide substitutions used to disrupt uORF-1 and uORF-2 (T → A) on splice donor and acceptor sites. Neither substitution is predicted to alter splicing sites.

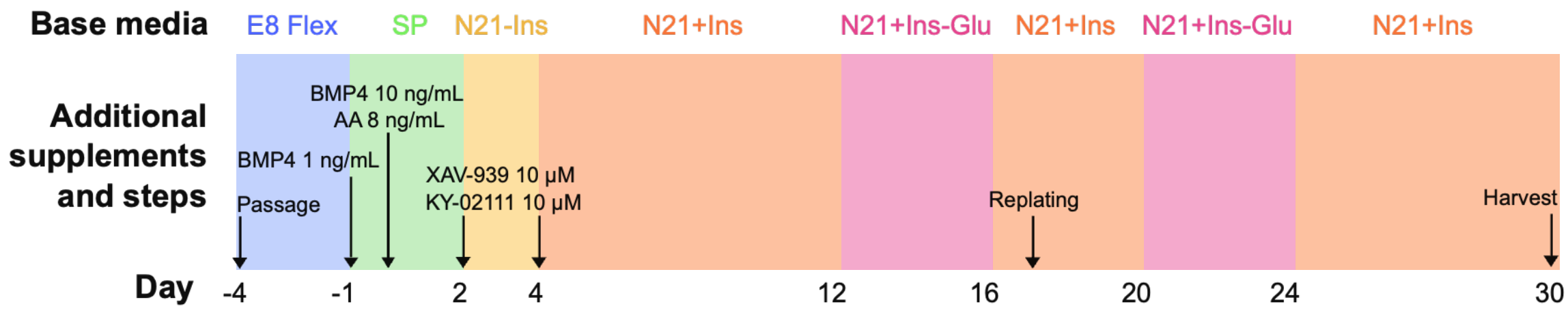

Figure S7: hiPSC-CM differentiation protocol.

hiPSCs were differentiated into hiPSC-CMs following a 30-day protocol. hiPSCs were passaged and maintained in E8 Flex, then transitioned through StemPro34 (SP), RPMI with N21-MAX Insulin Free Media Supplement (N21-Ins), RPMI with N21-MAX Media Supplement (N21+Ins), and RPMI medium with no glucose with N21-MAX Media Supplement (N21+Ins-Glu). Additional supplements were added including Bone Morphogenetic Protein 4 (BMP4), activin A (AA), and KY-02111 and XAV-939. Cells were replated on day 17 and harvested on approximately day 30.

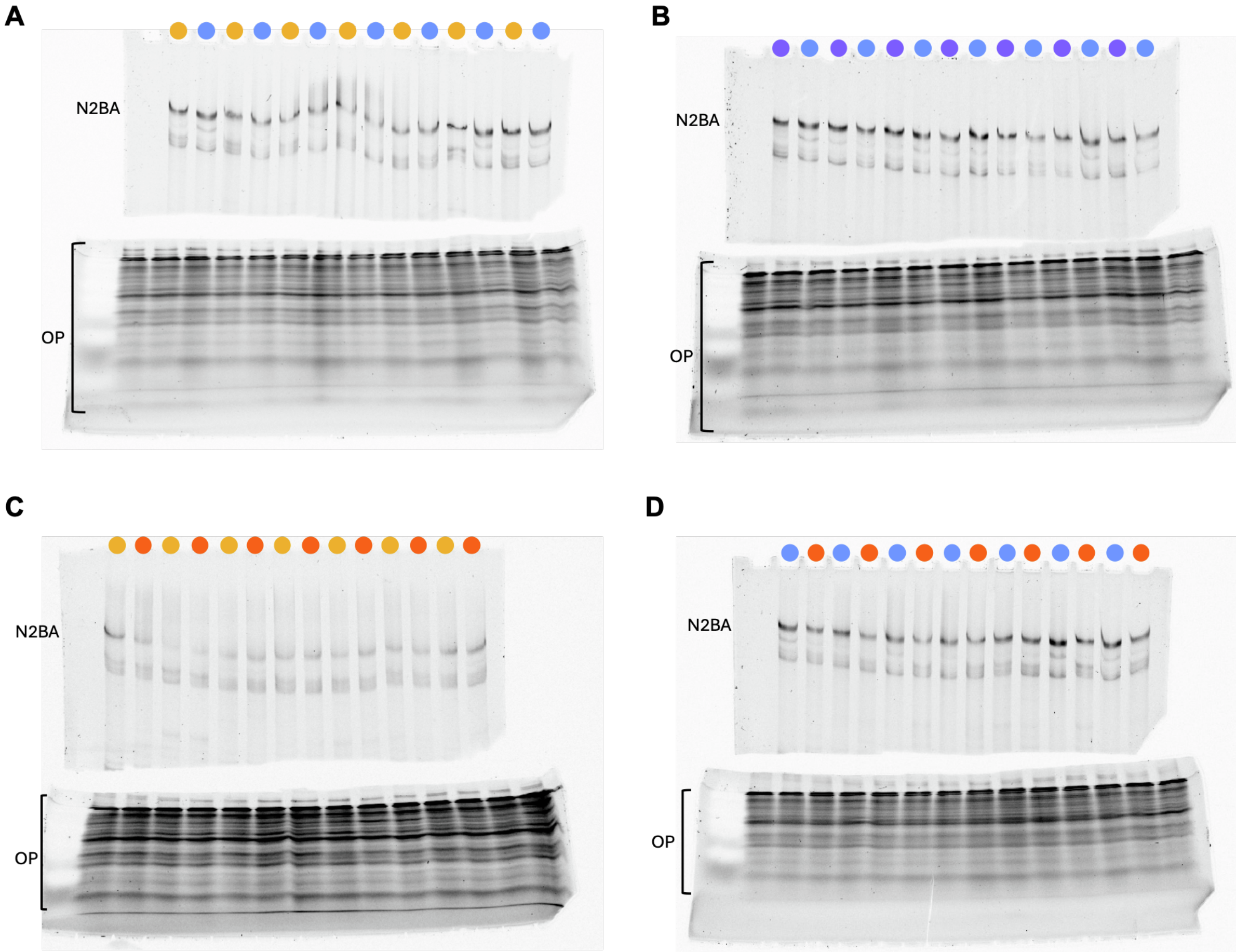

Figure S8: Uncropped versions of all gels shown in results.

Correspond to Figure 4D **(A)**, Figure 5A **(B)**, Figure 5B **(C)**, and Figure 5C **(D)**. Genotypes are colour coded – WT (blue), WT-ΔuORF (purple), *TTN*$^{+/-}$ (yellow), and *TTN*$^{+/-}$-ΔuORF (orange).
