# Supplementary material for: Functional analysis of TTN uORFs reveals context-dependent translational regulation
Source: Mol Ther Nucleic Acids. 2026 Aug 28;37(4):103077. doi: 10.1016/j.omtn.2026.103077 (PMC13586513; doi:10.1016/j.omtn.2026.103077)
Supplement: Supplementary file 2 [file mmc2.pdf]

Molecular Therapy
Nucleic Acids
Original Article

Functional analysis of *TTN* uORFs reveals context-dependent translational regulation

Rosemary B. Kirk,[1,2] Alexander J. Sparrow,[1] Marta Moya-Jódar,[1,2] Thomas C. Roberts,[3,4,5] Christopher N. Toepfer,[1,2] and Hugh Watkins[1]

[1]Division of Cardiovascular Medicine, Radcliffe Department of Medicine, University of Oxford, Oxford OX3 9DU, UK; [2]Oxford Organoid Hub, Sherrington Building, University of Oxford, Oxford OX1 3PT, UK; [3]Department of Paediatrics, University of Oxford, Oxford OX3 7TY, UK; [4]Institute of Developmental and Regenerative Medicine, University of Oxford, Oxford OX3 7TY, UK; [5]MDUK Oxford Neuromuscular Centre, Oxford OX3 7TY, UK

Familial dilated cardiomyopathy (DCM) is a common condition with a high clinical burden, and no therapies that target the underlying genetic mechanisms. The leading genetic cause of DCM is heterozygous truncating variants in *TTN*, which are thought to drive disease through haploinsufficiency, where titin is reduced. This raises the possibility of treating disease through therapeutic upregulation of endogenous *TTN* expression. Upstream open reading frames (uORFs) are regulatory elements that repress translation and have been targeted to upregulate gene expression in a range of genes. Two uORFs in the *TTN* 5' untranslated region have been shown to repress expression in luciferase reporter assays, suggesting potential for therapeutic targeting. Here, we investigated the role of *TTN* uORFs in reporter systems and in the endogenous locus in a human-induced pluripotent stem cell-derived cardiomyocyte (hiPSC-CM) model. While disruption of uORFs increased expression in luciferase reporter gene assays, including in hiPSC-CMs, CRISPR-Cas9-mediated disruption of uORFs at the endogenous *TTN* locus in hiPSC-CMs did not increase titin protein expression. These findings indicate that uORF-mediated repression of *TTN* is context-dependent and not recapitulated in a disease-relevant model. This highlights the complex regulation of *TTN* expression and the importance of validating regulatory elements in their endogenous context.

INTRODUCTION

Dilated cardiomyopathy (DCM) is a common condition with substantial clinical burden and no cure. DCM affects 1 in 250 people and is characterized by left ventricular dilatation, contractile dysfunction, and fibrosis, leading to complications ranging from heart failure to sudden cardiac death.[1,2] Current management is symptom-driven, and includes heart failure medications, implanted devices, and heart transplant.[3] DCM is familial in 30%–50% of cases, typically inherited in an autosomal dominant pattern with variable penetrance.[4] The leading genetic cause of DCM is heterozygous truncating variants in *TTN*, found in 18% of sporadic DCM and 25% of familial DCM.[5] *TTN* encodes titin, an exceptionally large 3–3.8 MDa protein that provides passive elasticity and contributes to organization and strain sensing in the cardiac sarcomere.[6] *TTN* truncating variants have traditionally been thought to drive disease through protein level haploinsufficiency[7]; however, emerging evidence shows that many variants also produce a persistent truncated protein product that is not degraded.[8,9] Despite this, current evidence supports haploinsufficiency as the key driver of disease in *TTN*-associated DCM,[9,10] and thus developing methods to increase titin levels is a therapeutic priority. While gene replacement therapies have reached clinical trials for other cardiomyopathies caused by loss-of-function variants, for example in the case of *MYBPC3* and *LAMP2*,[11,12] *TTN*'s large size precludes this approach due to viral vector packaging limits. In recent years, a variety of strategies to therapeutically upregulate gene expression to treat diseases driven by haploinsufficiency have emerged, raising the prospect of treating *TTN*-associated DCM by targeting this underlying disease mechanism.

Upstream open reading frames (uORFs) comprise a start codon in the 5' untranslated region (5' UTR) of an mRNA, running through to an in-frame stop codon, which may be within the 5' UTR or extend into the main coding sequence, i.e., the primary open reading frame (pORF). uORFs are present in over half of human mRNA transcripts and are associated with translational repression of the downstream pORF, reducing protein expression by 30%–80%.[13,14] As such, disrupting uORFs has emerged as a method of gene upregulation. Early studies used steric-blocking antisense oligonucleotides (ASOs) to target uORFs[15]; however, subsequent work has highlighted issues with the reproducibility of this approach.[16,17] A range of alternative uORF-targeting methods have since been developed. Protein upregulation was achieved in mice using an ASO that modulates RNA structure such that uORF translation is inhibited,[18] and in cell models by skipping uORF-containing exons,[19–21] or through direct disruption of uORFs by base editing or RNA editing.[21–23]

Received 2 June 2026; accepted 27 August 2026;
https://doi.org/10.1016/j.omtn.2026.103077.

Correspondence: Christopher N. Toepfer, John Radcliffe Hospital, Level 6, West Wing, Oxford OX3 9DU, UK.
E-mail: christopher.toepfer@cardiov.ox.ac.uk
Correspondence: Hugh Watkins, John Radcliffe Hospital, Level 6, West Wing, Oxford OX3 9DU, UK.
E-mail: hugh.watkins@rdm.ox.ac.uk

© 2026 The Authors.
Published by Elsevier Inc. on behalf of The American Society of Gene and Cell Therapy.
This is an open access article under the CC BY license (http://creativecommons.org/licenses/by/4.0/).

Previous studies have identified two highly conserved uORFs in the 5′ UTR of *TTN* transcripts, and have shown these to repress gene translation in luciferase reporter assays.[24,25] Both uORF start codons are in frame with the same stop codon within the 5′ UTR. The second uORF (uORF-2) has been shown to exert greater translational repression than the first (uORF-1), attributed to its stronger Kozak sequence context, while disruption of both uORFs simultaneously produces the greatest increase in reporter expression.[24] These studies support the notion that disrupting uORFs in the *TTN* gene could be a viable approach to increase titin protein levels in a therapeutic context. However, whether this uORF-mediated regulation is maintained in the native *TTN* gene context is currently unknown. This requires investigation both to advance understanding of *TTN* regulation, and to determine whether these uORFs are appropriate therapeutic targets for *TTN*-associated DCM. Here, we report an investigation of the repressive role of *TTN* uORFs, first using luciferase reporters in two cell models, then using gene-editing in the endogenous *TTN* locus in a human-induced pluripotent stem cell-derived cardiomyocyte (hiPSC-CM) model. We conclude that while the gene regulation mediated by the *TTN* uORFs is reproducible in a reporter gene context, including in hiPSC-CMs, this effect was not recapitulated for the endogenous gene in the disease-relevant cell model.

RESULTS

uORFs are present in key highly expressed *TTN* isoforms

There can be diversity in 5′ UTRs across distinct transcripts derived from the same gene locus due to alternative splicing and alternative transcription start site usage. This can affect whether a uORF is present. In addition, the various transcripts may contribute unequally to the overall mRNA pool. It was therefore necessary to confirm that the predominant *TTN* isoforms in adult humans contain the identified uORFs, thereby establishing them as potential therapeutic targets.

TTN comprises 15 Ensembl transcripts (Figure 1A), of which 10 share an identical 225 bp 5′ UTR, consisting of a 212 bp exon 1 and the first 13 bp of exon 2, after which the *TTN* pORF begins. These transcripts include the MANE select (matched annotation from the National Center for Biotechnology Information [NCBI] and the European Bioinformatics Institute [EMBL-EBI]) transcript (Ensembl: ENST00000589042.5; termed the "meta-transcript," a commonly used artificial reference transcript encompassing key canonical *TTN* exons), as well as major cardiac titin isoforms N2B, N2BA, and Novex-3.[26] This 225 bp 5′ UTR contains uORF-1 and uORF-2, both of which terminate at a shared stop codon within the 5′ UTR. The remaining five transcripts include two with 5′ UTRs of differing lengths that still contain both uORFs, and three transcripts that lack them. To our knowledge these latter transcripts are not linked to any known cardiac titin protein isoforms.

Exons 1 and 2 of the MANE Select transcript were assessed using publicly available ribosome profiling (Ribo sequencing, Ribo-seq) and RNA sequencing (RNA-seq) data (Figure 1B).[27,28] RNA-seq coverage across the uORF-containing region was comparable to, or greater than, that of the start of the pORF. Moreover, the highest peak in the initiating ribosome profile across the two exons localized to the uORF-2 start codon. This suggests active translation initiation at the uORFs, supporting functional relevance. Taken together, these data show that *TTN* transcripts containing the annotated uORF sequences constitute the majority of the total pool of mRNA generated from this locus, and that there is evidence for the translation of *TTN*-associated uORF sequences.

uORFs in the *TTN* 5′ UTR suppress translation in a luciferase reporter system in AD-293 cells

We next sought to experimentally confirm whether the identified uORFs function as translational repressors using a dual luciferase reporter system. The 225 bp uORF-containing 5′ UTR identified earlier was selected for investigation (Figure 2A). Previous work demonstrated that simultaneous disruption of both uORFs produces greater translational upregulation than disruption of either uORF individually,[24] a finding that we independently confirmed (Figure S1). Accordingly, subsequent experiments used two constructs in which this 5′ UTR was cloned upstream of a *Renilla* luciferase transgene, either in wild-type (WT) form or with both uORFs disrupted by mutation of their start codons (ΔuORF), enabling assessment of the maximal effect of uORF disruption. Start codons were mutated from ATG to AAG, previously reported to be one of the least efficient alternative start codons for initiating translation.[29] A separate firefly luciferase transgene served as an internal control.

These constructs were transfected into AD-293 cells (derived from HEK293), and luciferase activity was measured 24 h post-transfection. Disruption of both uORFs resulted in a 1.88-fold increase in luciferase expression ($p = 0.01$) (Figure 2B). This increase was not attributable to differences in mRNA abundance (Figure 2C). Together, these findings indicate that *TTN* uORFs can repress gene expression at the level of translation in this reporter system, consistent with previous reports.[24,25]

uORFs in the *TTN* 5′ UTR suppress translation in a luciferase reporter system in hiPSC-CMs

We next assessed whether uORFs in the *TTN* 5′ UTR exerted the same repression in a more physiologically relevant system. hiPSC-CMs were generated using a 30-days differentiation protocol,[30] yielding spontaneously contracting cells (data not shown). Expression of key cardiac genes and sarcomeric organization was confirmed by immunofluorescence staining (Figure 3A).

As transfection efficiency in hiPSC-CMs is typically low, conditions were first optimized using the dual luciferase reporter plasmid and a GFP-expressing plasmid. Following comparison of several transfection reagents, a protocol was selected that produced robust GFP expression, with GFP-positive area comprising approximately 8% of the imaged hiPSC-CM area (Figure 3B), together with robust unnormalized firefly luciferase activity within the lower end of the range observed in AD-293 cells under the same assay conditions (Figure S2). The dual-luciferase design also minimized the impact of variation in transfection efficiency, as *Renilla* luciferase activity

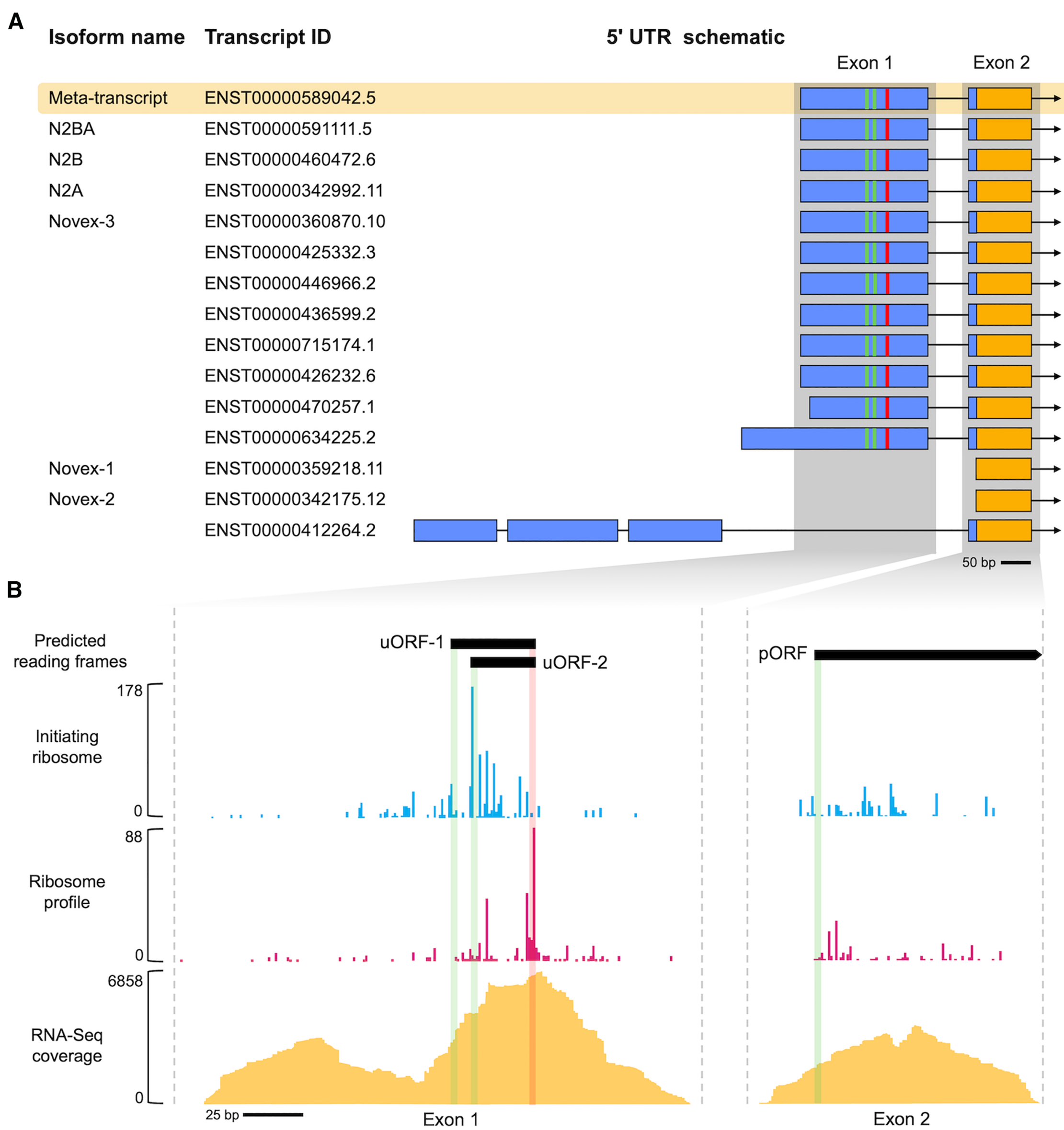

Figure 1. A uORF-containing *TTN* 5′ UTR is highly expressed and exhibits strong uORF ribosome occupancy

(A) Of the 15 *TTN* isoforms, 12 (including key cardiac isoforms) contain the two identified uORFs in their 5′ UTR. The MANE Select transcript is highlighted. The 5′ UTR is shown in blue, the pORF in yellow, uORF start codons in green, and the shared uORF stop codon in red. Exon lengths are shown to scale (scale bar, 50 bp); introns are not to scale. Exons 1–2 of 364 are displayed. (B) Publicly available ribosome profiling (Ribo-seq) and RNA sequencing (RNA-seq) data accessed from the genome-wide information on protein synthesis (GWIPS)-viz browser for *TTN* exons 1 and 2. Highlighted uORF start codons correspond to prominent ribosome initiation peaks, with stronger signal at uORF-2 compared to the pORF start codon (scale bar, 25 bp).

was normalized to the firefly luciferase signal from the same reporter construct.

The *TTN* 5′ UTR dual luciferase reporter constructs were then transfected into hiPSC-CMs and luciferase activity measured 24 h post-transfection. Disruption of both uORFs resulted in a 1.77-fold increase in luciferase expression (p = 0.011) (Figure 3C) that could not be explained by altered mRNA transcript levels (Figure 3D). These findings were consistent with results in AD-293 cells, suggesting that the ability of uORFs to exert repression on translation is maintained in the cardiac cell environment.

Engineering hiPSC-CMs with disruption of uORFs in the *TTN* 5′ UTR

To determine whether uORF-mediated repression observed in reporter assays is recapitulated at the endogenous *TTN* locus, we

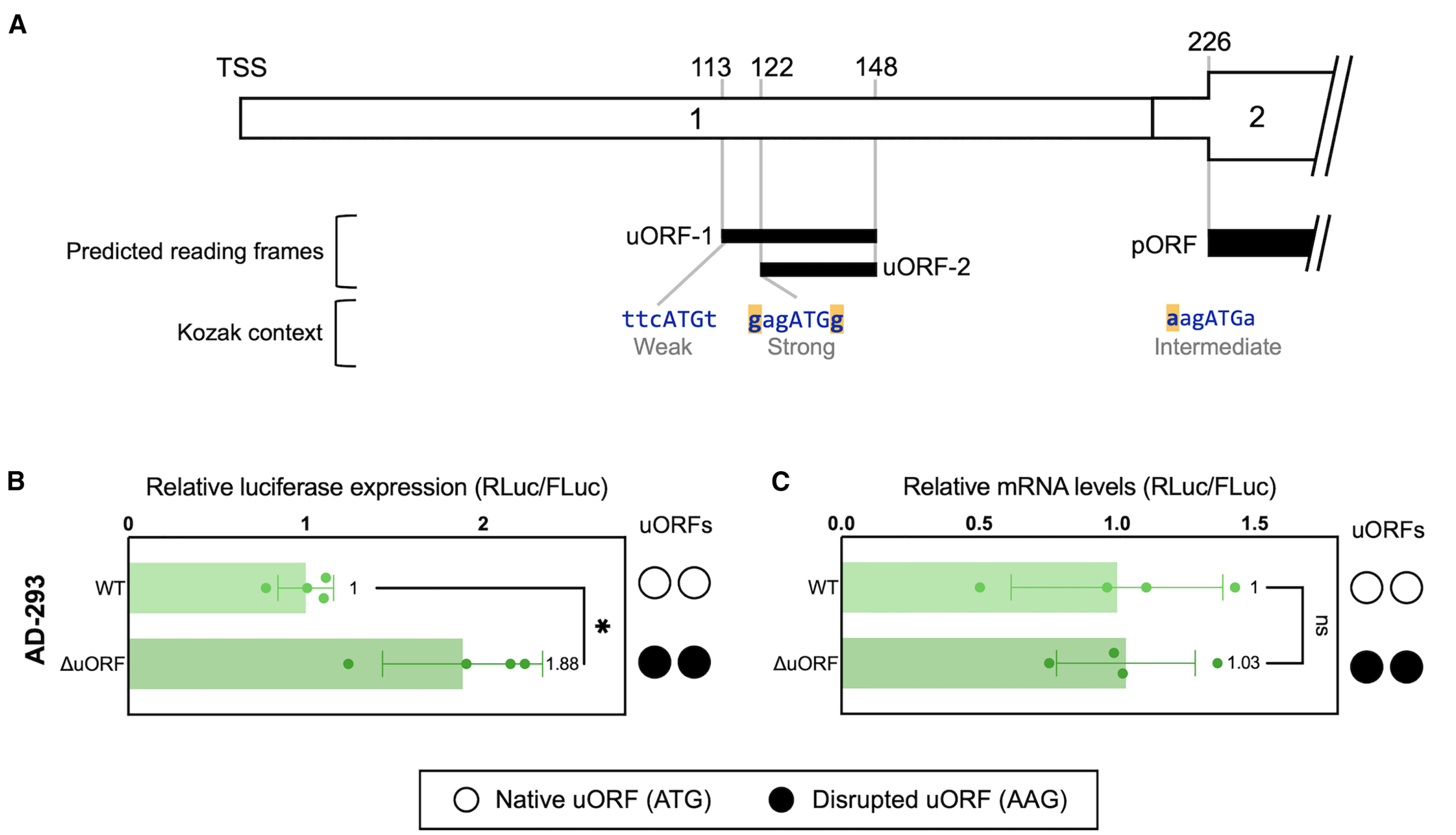

Figure 2. uORFs in the *TTN* 5′ UTR exert translational repression in a dual luciferase reporter assay in AD-293 cells

(A) Schematic of the predominant 225 bp *TTN* 5′ UTR containing two predicted uORFs (uORF-1 and uORF-2). Kozak context of each start codon is shown, with features contributing to whether the Kozak context is strong highlighted. (B) AD-293 cells were transfected with *TTN* 5′ UTR dual luciferase reporter constructs containing the *TTN* 5′ UTR in either wild-type form (WT) or with both uORFs disrupted (ΔuORF). Luciferase activity was measured 24 h post-transfection. (C) AD-293 cells were transfected as in (B) and harvested 24 h post-transfection for analysis of *Renilla* and firefly luciferase mRNA levels by RT-qPCR. Bars represent mean (SD); $n = 4$ biological replicates, each containing 4 technical replicates (B) or 2 technical replicates (C). Values were normalized such that the mean of the WT control group was returned to a value of 1. Statistical significance was determined by performing an unpaired two-sided t test. *$p = 0.01$; ns, not significant.

generated hiPSC-CMs in which uORF start codons were disrupted by CRISPR-Cas9 gene-editing.

Prior to genome editing, hiPSC-CM RNA-seq data were analyzed to confirm expression of *TTN* transcripts with uORF-containing 5′ UTRs. Two hiPSC-CM lines were assessed: one WT and one harboring a known disease-causing heterozygous truncating *TTN* variant, NM_001267550.2:c.71602C>T ($TTN^{+/-}$), both derived from the same hiPSC parental line. Across both genotypes, the vast majority of transcripts contained uORF-containing 5′ UTRs, comprising 99.3% ± 2.55% of transcripts in WT cells and 99.3% ± 2.12% in $TTN^{+/-}$ cells (mean ± SD) (Figure 4A). Of these, transcripts containing the 225 bp 5′ UTR used in the luciferase assays were the most abundant, accounting for 65.5% ± 15.0% of total transcripts in WT cells and 58.8% ± 4.28% in $TTN^{+/-}$ cells. These findings support the suitability of this model for assessing the impact of uORF disruption.

A CRISPR-Cas9 homology-directed repair (HDR) strategy was designed to mutate the start codons of both uORF-1 and uORF-2 from ATG to AAG as previously tested by luciferase assay (Figure 4B). Both uORFs were disrupted simultaneously to investigate the maximal possible effect of uORF disruption. Gene editing was performed in both WT and $TTN^{+/-}$ hiPSC lines, yielding clonal lines with homozygous disruption of both uORFs (ΔuORF) as well as CRISPR control clones without the intended mutation. This resulted in four cell lines: WT, WT-ΔuORF, $TTN^{+/-}$, and $TTN^{+/-}$-ΔuORF. Successful editing was confirmed by Sanger sequencing (Figure 4C). Sequencing of the surrounding area confirmed the presence of nearby heterozygous nucleotides, excluding monoallelic disruption (Figure S3).[31] All cell lines maintained expression of pluripotency markers (Figure S4), showed no karyotypic abnormalities (Figure S5), and exhibited no predicted off target mutations (Table S1).

Titin protein levels were quantified in CRISPR control lines (WT and $TTN^{+/-}$). Samples were resolved using a two-phase sodium dodecyl sulfate (SDS)-polyacrylamide gel. Due to its exceptionally large molecular mass, titin was retained in the low-percentage upper phase, while total other cardiac proteins (“other protein,” OP) were quantified from the higher-percentage lower phase. Expression of the predominant hiPSC-CM titin isoform, N2BA, was normalized to OP.

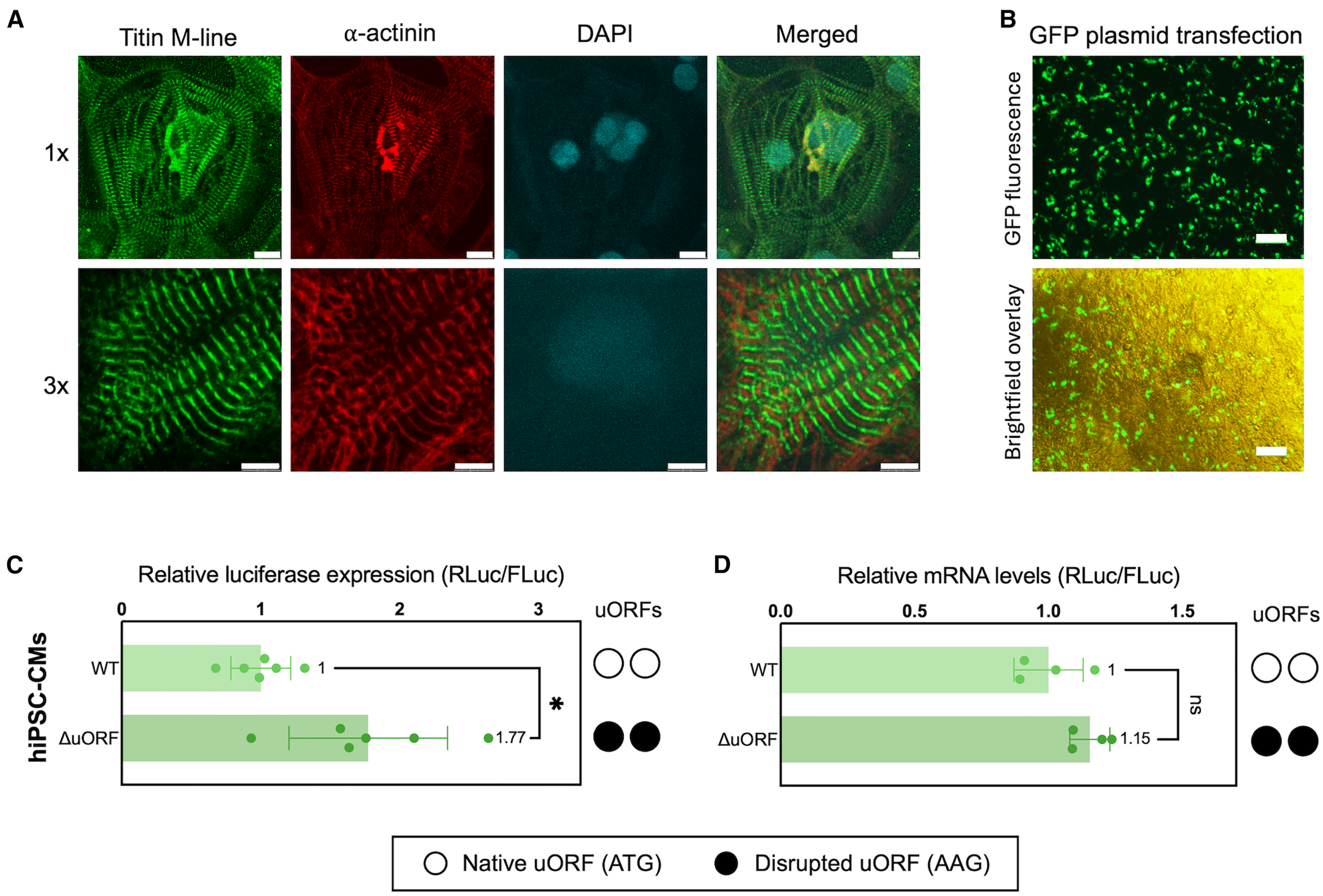

Figure 3. uORFs in the *TTN* 5' UTR exert translational repression in a dual luciferase reporter in hiPSC-CMs

(A) A 30-days differentiation protocol produced hiPSC-CMs with expression of key sarcomeric proteins (e.g., Titin and α-actinin) and visible sarcomeres. Images acquired with a 63× oil immersion objective with 1× or 3× magnification (scale bars: 1×, 10 μm; 3×, 5 μm). (B) Representative image of a transfection of hiPSC-CMs with a GFP-expressing plasmid using the optimized transfection conditions used for dual luciferase reporter transfection. GFP-positive area comprised approximately 8% of the imaged hiPSC-CM area under the selected transfection conditions (scale bars, 100 μm). (C) hiPSC-CMs were transfected with *TTN* 5' UTR dual luciferase reporter constructs containing the *TTN* 5' UTR in either wild-type form (WT) or with both uORFs disrupted (ΔuORF). Luciferase activity was measured 24 h post-transfection. (D) hiPSC-CMs were transfected as in (C) and harvested 24 h post-transfection for analysis of *Renilla* and firefly luciferase mRNA levels by RT-qPCR. Bars show mean (SD), $n = 6$ biological replicates each containing 4 technical replicates for (C) and $n = 4$ biological replicates each containing 2 technical replicates for (D). Values were normalized such that the mean of the WT control group was returned to a value of 1. Statistical significance was determined by performing an unpaired two-sided *t* test. $^{*}p < 0.05$; ns, not significant.

This analysis confirmed haploinsufficiency of N2BA in $TTN^{+/-}$ cells (60.3% of WT levels, $p = 0.033$) (Figures 4D and 4E).

Together, these data establish hiPSC-CMs as a suitable system for alteration of endogenous *TTN* uORFs, in which changes in titin expression can be detected.

Disruption of *TTN* uORFs in their native genomic context in hiPSC-CMs does not increase titin protein expression

To determine whether uORF disruption in the endogenous *TTN* locus enhances titin protein expression, titin levels were quantified in CRISPR-edited hiPSC-CMs. Unexpectedly, in both WT and $TTN^{+/-}$ cells, homozygous disruption of both uORFs did not result in a significant increase in full-length titin protein expression compared to unedited controls (Figures 5A and 5B). This was further supported by direct comparison of $TTN^{+/-}$-ΔuORF cells with WT cells, which demonstrated significantly reduced full-length titin (64.8% of WT levels, $p = 0.0019$) (Figure 5C). This is comparable to the titin levels observed in unedited $TTN^{+/-}$ cells compared to WT cells (Figures 4D and 4E), indicating no rescue of titin levels.

To assess whether these findings could be explained by changes in transcript abundance, *TTN* mRNA levels were quantified by quantitative reverse-transcription PCR (RT-qPCR). No significant differences in *TTN* mRNA levels were observed following uORF disruption (Figure 5D).

Disruption of *TTN* uORFs is not predicted to alter expression or regulation via changes to mRNA secondary structures, splicing, or binding motifs

The discrepancy between uORF-mediated repression observed in luciferase reporter assays and the lack of effect at the endogenous

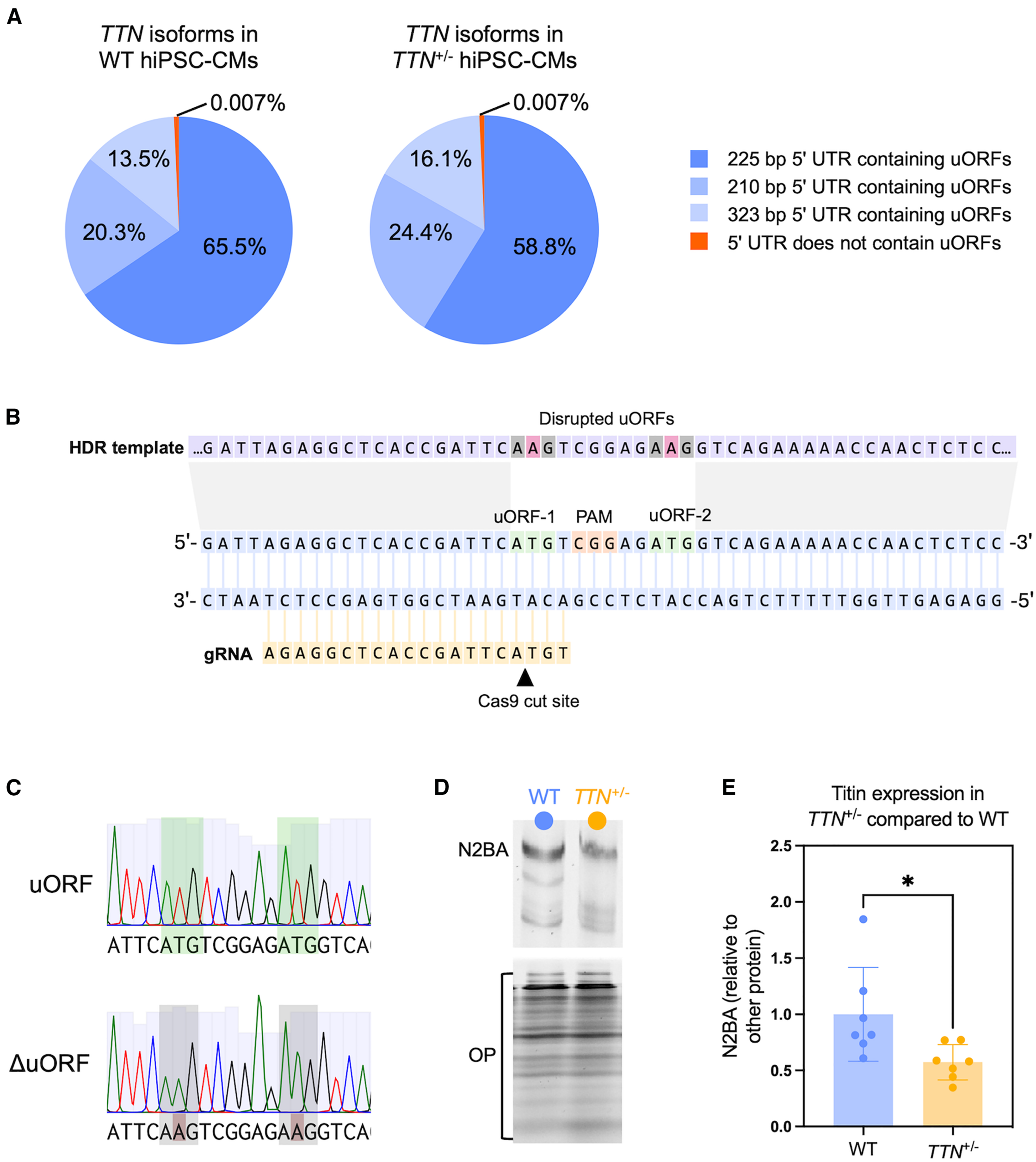

Figure 4. Generation and validation of hiPSC-CMs with CRISPR-mediated disruption of *TTN* uORFs

(A) RNA-seq analysis of *TTN* isoform expression in wild-type (WT) and *TTN* variant carrying ($TTN^{+/-}$) hiPSC-CMs showing that the majority of transcripts incorporate uORF-containing 5' UTRs. $n = 4$ biological replicates for $TTN^{+/-}$ and 5 biological replicates for WT. Isoform usage was calculated as the proportion of total gene-level transcripts per million (TPM) contributed by each transcript within each sample. (B) Schematic of the CRISPR-Cas9 homology-directed repair (HDR) strategy used to mutate the start codons of uORF-1 and uORF-2 (ATG to AAG). Gene shown in 5' to 3' orientation; located on negative strand in the genome. (C) Representative Sanger sequencing traces confirming homozygous disruption of both *TTN* uORFs (ΔuORF). (D) Representative lanes from a two-phase SDS-polyacrylamide protein gel stained with SYPRO Ruby showing the full-length titin isoform N2BA (upper phase), with other protein (OP; lower phase) used for normaliztion, in CRISPR-control hiPSC-CMs that were wild-type (WT, blue) or harboring a heterozygous truncating *TTN* variant ($TTN^{+/-}$, yellow). (E) Quantification of N2BA protein relative to OP from the gel in (D). Bars represent mean (SD); $n = 7$ independent differentiations per genotype. Values were normalized such that the mean of the WT group was set to 1. Statistical significance was assessed using an unpaired t test. $^*p < 0.05$.

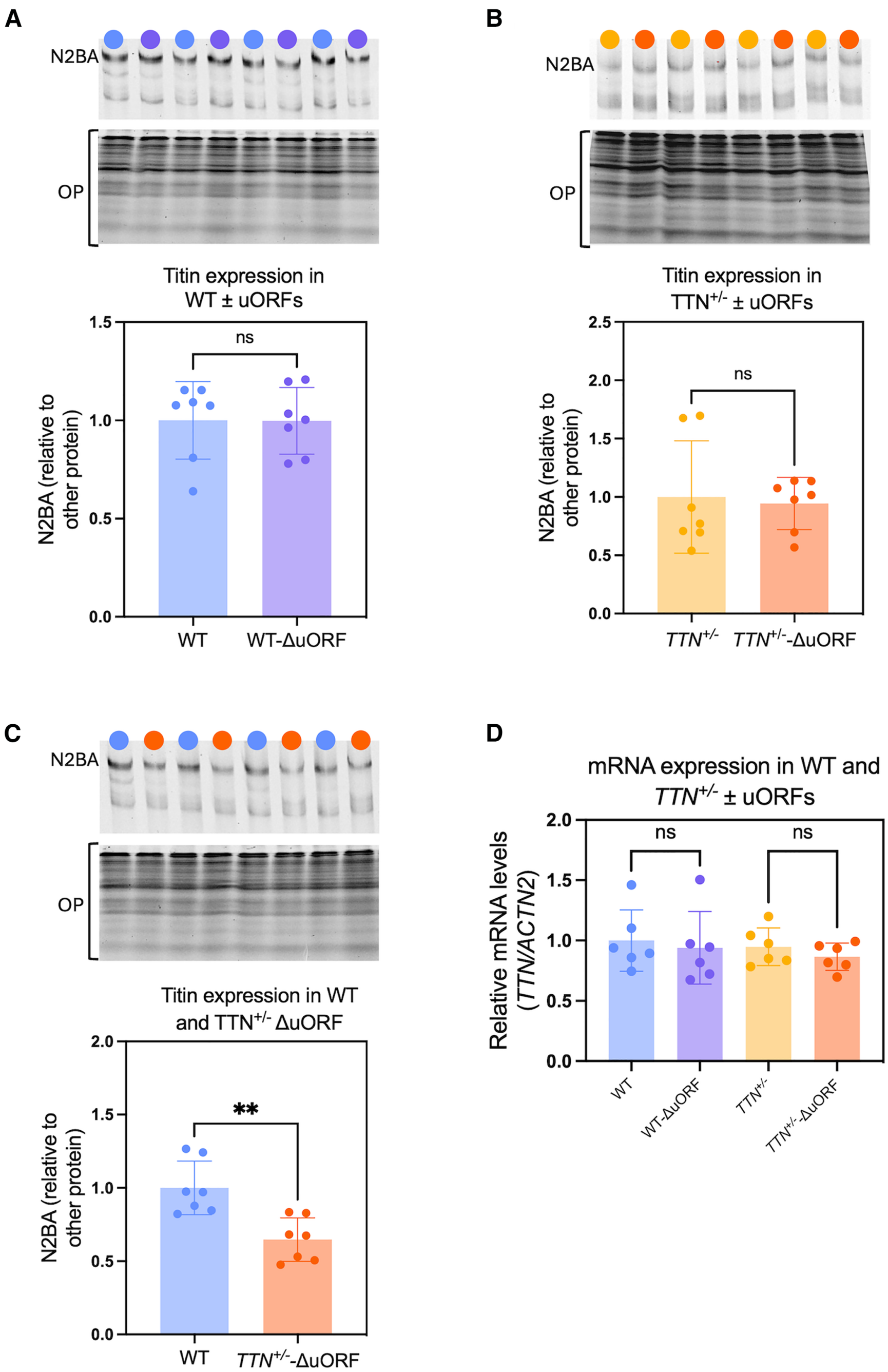

Figure 5. CRISPR-mediated disruption of *TTN* uORFs does not increase titin protein expression

(A) Representative lanes and quantification from a two-phase SDS-polyacrylamide protein gel stained with SYPRO Ruby measuring the full-length titin isoform N2BA (upper phase), normalized to other protein (OP; lower phase) in wild-type hiPSC-CMs (WT, blue) and uORF-disrupted cells (WT-ΔuORF, purple). (B) Representative lanes and quantification of N2BA protein relative to OP in *TTN* truncation-background hiPSC-CMs ($TTN^{+/-}$, yellow) and uORF-disrupted cells ($TTN^{+/-}$-ΔuORF, orange). (C) Representative lanes and quantification of N2BA protein relative to OP in $TTN^{+/-}$-ΔuORF hiPSC-CMs relative to WT hiPSC-CMs demonstrates no rescue of haploinsufficiency following uORF disruption. (D) *TTN* mRNA levels in hiPSC-CMs quantified by RT-qPCR relative to *ACTN2*. Bars represent mean (SD); $n = 7$ independent differentiations per genotype for (A–C) and $n = 6$ for (D). Values were normalized such that the mean of the WT group was set to 1. Statistical significance was assessed using Mann-Whitney tests (A and B) or unpaired two-sided *t* tests (C) for two-group comparisons, and one-way ANOVA for multiple comparisons (D). $^{**}p < 0.01$; ns, not significant.

TTN locus raised the possibility that sequence context or the introduced mutation might reduce *TTN* expression through alternative mechanisms, thereby masking any increase in translation resulting from uORF disruption.

RNA-fold was used to predict secondary structures for the *TTN* 5′ UTR followed by either the start of *TTN* or *Renilla* luciferase (400 bp total analyzed), in both WT and uORF-disrupted configurations (Figure S6A).[32] No major structural changes were predicted; instead, the uORF region showed higher unpaired probability when followed by *TTN*, consistent with accessibility to ribosomes and the potential to exert translational repression. In addition, the predicted secondary structures in the region of the pORF start codon were unchanged with alteration of uORF start codons from AUG to AAG, suggesting that this change is not predicted to impair pORF translation. In the *TTN* context, disruption of the uORFs was predicted to introduce an additional local hairpin within the uORF region; however, this structure had a low predicted base-pairing probability and is therefore unlikely to exert a substantial repressive effect on translation.

The *TTN* 5′ UTR was analyzed for RNA-binding motifs using BRIO (BEAM RNA interaction motifs).[33] No motifs were predicted to be disrupted by uORF mutagenesis (Figure S6B). Finally, Human Splicing Finder analysis indicated no predicted impact of uORF disruption on splicing (Figure S6C).[34]

Collectively, these analyses do not suggest any effects on RNA structure, RNA-binding motifs, or splicing that would be expected to reduce *TTN* expression and thereby mask translational de-repression following uORF disruption.

DISCUSSION

In this study, we investigated the regulatory role of uORFs in the 5' UTR of *TTN* and their potential as therapeutic targets to enhance *TTN* expression. Consistent with previous reports,[24,25] we demonstrate that *TTN* uORFs repress translation in luciferase reporter assays in heterologous cell lines (Figure 2B). uORF-regulation of reporter constructs was maintained when these were transfected in hiPSC-CMs indicating that the cardiomyocyte environment does not preclude uORF-mediated regulation *per se* (Figure 3C). However, in contrast to these findings, CRISPR-Cas9-mediated disruption of both uORFs at the endogenous *TTN* locus did not result in increased titin protein expression in hiPSC-CMs (Figures 5A–5C). These data indicate that while the *TTN* uORFs possess repressive capacity, this effect is not observed in their native genomic context. This discrepancy highlights both the complex regulation of *TTN*, and the limitations of simplified reporter systems in modeling endogenous gene regulation.

Several potential explanations for the discrepancy between uORF-mediated repression in luciferase assays and the lack of effect at the endogenous *TTN* locus were considered. One possibility was that differences in cellular context could alter uORF translation; recent evidence suggests that the relative use of translation initiation sites of different strengths varies between dividing cells (e.g., AD-293 cells) and non-dividing cells (e.g., hiPSC-CMs).[35] However, translational repression was observed at a similar magnitude in luciferase assays performed in both AD-293 cells and hiPSC-CMs, arguing against the observed discrepancy resulting from cell type-specific effects (Figures 2B and 3C). Alternatively, the lack of uORF-mediated repression in hiPSC-CMs could reflect preferential expression of *TTN* isoforms lacking uORFs; however, this was not supported by RNA-seq analysis (Figure 4A). A further possibility is the presence of feedback mechanisms maintaining titin homeostasis. However, while this could account for the absence of upregulation in the WT cells, increased expression would be expected to be permitted in the haploinsufficient $TTN^{+/-}$ model, which was not observed (Figure 5B).

Another possibility was that mutagenesis of uORF start codons in their endogenous genomic context introduced unintended effects on *TTN* expression, either preventing uORF-mediated repression or offsetting any translational de-repression resulting from uORF disruption. However, there was no evidence from our data or predictive analysis to support this. *TTN* mRNA levels were unchanged following uORF disruption, arguing against an effect on mRNA stability (Figure 5D). *In silico* analyses did not predict substantial alterations in RNA secondary structure (Figure S6A). Although disruption of the uORFs in the *TTN* context was predicted to introduce an additional local hairpin structure, this had a low predicted base-pairing probability. Importantly, the uORF region was still predicted to be accessible within the *TTN* sequence, and the local structural environment of the pORF start codon was unchanged. Finally, there were no predicted alterations to RNA-binding motifs or splicing signals (Figures S6B and S6C). These analyses have limitations; they are static predictions, rely on simplified models, and do not factor in cell type. Further, RNA structure analyses were restricted to a 400 nt region, capturing the local structural environment but not long-range interactions across the full transcript. These predictive tools also do not exclude other untested mechanisms of post-transcriptional regulation. Overall, these analyses do not suggest a clear alternative mechanism suppressing *TTN* expression in the uORF-disrupted cells.

A more likely explanation is that the regulatory impact of uORFs is dependent on the sequence context and other regulators of gene expression. In reporter systems, the 5' UTR is upstream of a short, heterologous coding sequence, whereas the *TTN* transcript is exceptionally large and likely subject to additional layers of regulation. In this setting, uORF-mediated repression may not be a dominant mechanism controlling translation, or may be mitigated by downstream constraints on translational efficiency. Thus, the lack of increased titin expression following uORF disruption may reflect broader regulatory constraints rather than a failure of uORFs to function as repressive elements. Identifying specific sequence features that influence translational efficiency in this context would be technically challenging given the size and complexity of *TTN*, and may not yield therapeutically tractable targets.

These findings have important implications for therapeutic strategies aimed at increasing *TTN* expression. Our approach, of generating cell lines with disruption of both uORFs, was designed to assess the maximal possible translational upregulation that uORF disruption could achieve prior to consideration of more clinically relevant methods such as ASOs or base editing. The absence of increased titin expression in this context suggests that uORF-targeted approaches are unlikely to achieve meaningful upregulation of *TTN* expression in DCM.

A range of other genetic therapy strategies have been explored in models of *TTN*-associated disease. These include variant-specific approaches; levels of full-length titin have been rescued by genetic correction of a variant,[9] or by skipping a 17 kb exon, where many DCM-causing *TTN* variants are located.[36] However, given that pathogenic *TTN* variants are distributed across the length of the gene, each individual gene editor or ASO would be applicable to a limited pool of patients with actionable variants, limiting broader translatability. In contrast, mutation-agnostic strategies that increase overall *TTN* expression may have broader therapeutic potential. Titin levels have been successfully increased in hiPSC-CM models by using CRISPR activation (CRISPRa), which activates transcription,[10] and by use of the proteasome inhibitor MG132 to block protein degradation by the ubiquitin proteasome system.[9] A further study reported an improvement in DCM phenotype by knockdown of the predominant cardiac antisense transcript of *TTN* (*TTN-AS1-276*), leading to preferential expression of a more compliant cardiac *TTN* isoform.[37] Together with our findings, these studies suggest that *TTN* expression

may be more dependent on transcript abundance and protein stability than on translation initiation.

Beyond implications for *TTN*, our findings have broader relevance for the study of uORFs and other mechanisms of 5' UTR-mediated regulation. Luciferase reporter assays are widely used to assess the repressive capacity of uORFs and other regulatory elements; however, our results demonstrate that effects observed in such systems do not necessarily translate to the endogenous genomic context. While several studies have validated 5' UTR-targeting strategies in both reporter systems and endogenous models,[18,21,23,38–40] many others rely predominantly on luciferase assays or computational prediction.[24,25,41–43] While the discrepancy observed here may reflect features specific to *TTN*, given its exceptional size and complex regulatory architecture, it remains unknown how broadly these findings apply. Our findings suggest that the contribution of uORFs to gene regulation should be evaluated on a gene-by-gene basis and underscore the importance of validating luciferase assay findings in their native genomic context.

Several limitations of our work should be considered. Although hiPSC-CMs provide a relevant human cardiac model, they may not fully recapitulate the maturation state and translational regulation of adult cardiomyocytes. In addition, both uORFs were disrupted simultaneously at the endogenous *TTN* locus to investigate the maximal possible effect of uORF disruption. While individual disruption of each uORF was examined in the reporter assays (Figure S1), their individual contributions to endogenous *TTN* regulation remain unknown. We also cannot exclude residual translation initiation at the endogenous uORFs following mutation of the uORF start codons, as this study did not directly assess translational dynamics such as ribosome occupancy or initiation rates at the endogenous locus. While the same mutations reproducibly relieved translational repression in reporter assays, future studies employing approaches such as ribosome profiling could provide additional mechanistic insight into endogenous uORF translation. This study assessed steady-state protein levels, which was considered the most relevant endpoint for therapeutic upregulation. However, uORF disruption could still influence translation during dynamic changes in gene expression that were not captured here. Although our assay sensitivity was sufficient to detect *TTN* haploinsufficiency, more modest increases in titin expression may not have been detectable. However, the observation that both $TTN^{+/-}$ and $TTN^{+/-}$-ΔuORF cells exhibited similarly reduced full-length titin protein levels compared to WT cells (60.3% and 64.8%, respectively; Figures 4E and 5C) suggests that any such increases were minimal and unlikely to be therapeutically meaningful. Finally, *in silico* analyses of RNA secondary structure, RNA-binding protein motifs, and splicing are inherently limited. Secondary structure predictions were restricted to local sequence regions due to computational constraints and represent ensembles of possible conformations rather than a single defined structure. Moreover, these models do not capture dynamic RNA folding *in vivo* or interactions with RNA-binding proteins.

In summary, this study demonstrates that *TTN* uORFs, while capable of repressing translation in reporter systems, do not measurably regulate titin protein expression at the endogenous locus in hiPSC-CMs. These findings underscore the importance of studying regulatory elements in their native genomic context and highlight the challenges of translating uORF-targeting strategies into therapeutic approaches for complex genes such as *TTN*.

MATERIALS AND METHODS

Bioinformatics and public datasets

Details of *TTN* isoforms were obtained from Ensembl.[44] Publicly available human Ribo-seq data and RNA-seq data were obtained from and visualized on the GWIPS-viz browser.[27,28] RNA-fold was used for RNA secondary structure visualization.[32] BRIO was used to analyze sequences for binding motifs.[33] The Human Splicing Finder sequence analysis tool was used to assess the effects of introduced variants on splicing signals.[34]

Reporter plasmid generation

The dual luciferase reporter plasmid for testing the translational activity of 5' UTRs has been described previously.[14,16,21] The *TTN* 5' UTR was inserted upstream of the *Renilla* luciferase (RLuc) transgene cassette by Gibson assembly using the NEBuilder HiFi DNA Assembly Cloning Kit (New England Biolabs, Ipswich, MA, USA). A separate firefly luciferase (FLuc) cassette acted as an internal control.

AD-293 cell culture and transfection

AD-293 cells (Agilent Technologies, Santa Clara, CA, USA), HEK293-derived cells with improved cell adherence properties, were cultured in medium comprising Dulbecco's modified Eagle's medium (DMEM) D6546 (Sigma-Aldrich, St. Louis, MO, USA) that was supplemented with 10% fetal bovine serum (FBS) (Merck KGaA, Darmstadt, Germany), 1% L-glutamine (Merck KGaA, Darmstadt, Germany), and 1% pen-strep (Gibco, Thermo Fisher Scientific, Waltham, MA, USA).

For reporter construct luciferase assays, AD-293 cells were seeded at 2×10^4 cells per well in a 96-well plate. At 24 h, they were transfected with 100 ng of vector per well using 0.1 μL of Lipofectamine 2000 (Thermo Fisher Scientific, Waltham, MA, USA). Plasmid and Lipofectamine 2000 were diluted separately in Opti-MEM (Gibco, Thermo Fisher Scientific, Waltham, MA, USA), then combined and incubated for 20 min before dropwise addition to cells. For reporter construct mRNA assays the same process was followed, instead using 1×10^5 cells per well in a 24-well plate, transfected with 500 ng of vector and 0.5 μL of Lipofectamine 2000.

hiPSC-CM culture and transfection

KOLF2 hiPSCs (HPSI0114i-kolf-2, ECACC 77650100) were cultured in Essential 8 (E8) Flex medium on GelTrex coated six-well plates (both Gibco, Thermo Fisher Scientific, Waltham, MA, USA). Cells were incubated at 37°C in 5% CO_2 with medium changes every second day. Passages were performed when cells were 60%–80%

confluent and cells maintained in basal medium containing 10 μM ROCK inhibitor Y-27632 (Selleck Chemicals, Houston, TX, USA) for 24 h after passage.

hiPSCs were differentiated into hiPSC-CMs following a 30-days protocol as previously described, and summarized in Figure S7.[30] In brief, when hiPSCs were 50%–80% confluent, medium was changed to StemPro34 (Gibco, Thermo Fisher Scientific, Waltham, MA, USA) supplemented with 1 ng/mL Bone Morphogenetic Protein 4 (BMP4) for 24 h, then to StemPro34 supplemented with 10 ng/mL BMP4 and 8 ng/mL activin A for 48 h (all supplements Miltenyi Biotec, Bergisch Gladbach, Germany). Medium was then changed to RPMI (Thermo Fisher Scientific, Waltham, MA, USA) with N21-MAX Insulin Free Media Supplement (R&D Systems, Bio-Techne, Minneapolis, MN, USA) and 10 μM KY-02111 and 10 μM XAV-939 (both Tocris, Bio-Techne, Bristol, UK) for 48 h, then to RPMI supplemented with N21-MAX Media Supplement (R&D Systems, Bio-Techne, Minneapolis, MN, USA) containing KY-02111 and XAV-939 for 48 h. Cells were subsequently transitioned to RPMI with N21-MAX Media Supplement with no additional supplements. They were maintained in this with medium changes every 2 days. All differentiations underwent two periods of glucose deprivation using RPMI medium with no glucose (Thermo Fisher Scientific, Waltham, MA, USA) to select for survival of cardiomyocytes over other cell types. All differentiations were replated on day 17 of the protocol. Cells were harvested on day 30 ± 2 days for downstream assays.

For reporter construct luciferase assays, hiPSC-CMs were replated at 7.5×10^4 cells per well in a 96-well plate. At 72 h, they were transfected with 100 ng of vector per well using 0.3 μL of ViaFect (Promega, Madison, WI, USA). ViaFect and plasmid were diluted together in Opti-MEM and incubated for 20 min before dropwise addition to cells. For reporter construct mRNA assays the same process was followed, instead using 3.75×10^5 cells per well in a 24-well plate, transfecting with 500 ng of vector and 1.5 μL of ViaFect.

Dual luciferase assay

Luciferase activity was determined using the Dual-Glo Luciferase kit (Promega, Madison, WI, USA) following the manufacturer's protocol. Opaque white 96-well plates were used for the assay to reduce luminescence cross-talk between wells, and FLuc and RLuc activity were measured using the FluoSTAR Omega (BMG LABTECH, Ortenberg, Germany). For each experiment, at least four independent biological repeats were performed, each containing four technical replicates. RLuc values were normalized to FLuc values.

RT-qPCR

mRNA was extracted using the RNeasy Mini Kit (QIAGEN, Hilden, Germany). Cells were harvested into 300 μL of RLT buffer containing 3 μL of β-mercaptoethanol (Sigma-Aldrich, St. Louis, MO, USA) and extraction performed as per the manufacturer's instructions. cDNA was generated using the High-Capacity cDNA Reverse-Transcription Kit (Thermo Fisher Scientific, Waltham, MA, USA) according to manufacturer's instructions using 100–500 ng input total RNA.

For quantitative polymerase chain reaction (qPCR) amplification of FLuc and RLuc mRNA, 1 μL of cDNA was combined with 5 μL 2× SYBR Green PCR Master Mix (QIAGEN, Hilden, Germany), 0.05 μL of ROX reference dye (QIAGEN, Hilden, Germany), 0.7 μL of each primer (at 0.3 μM concentration), and 2.55 μL of RNase-free water. Samples were run in duplicate using the QuantStudio 5 Real-Time PCR system (Applied Biosystems, Thermo Fisher Scientific, Waltham, MA, USA), and in each sample both FLuc and RLuc transcripts were measured and RLuc quantified relative to FLuc. Reaction specificity was confirmed by post-run melting curve analysis. Primer sequences are provided in Table S2.

For qPCR of *TTN* mRNA, 1 μL of cDNA was combined with 5 μL of TaqMan Fast Universal PCR Master Mix (2×), 0.5 μL each of a VIC and FAM TaqMan probe, and 3 μL of nuclease-free water (all TaqMan products Applied Biosystems, Thermo Fisher Scientific, Waltham, MA, USA). mRNA expression of *TTN* (FAM probe) was quantified relative to a cardiomyocyte-appropriate housekeeper *ACTN2* (VIC probe) using the QuantStudio 5 Real-Time PCR system, with samples run in duplicate. Details of TaqMan probes used are provided in Table S3.

Immunofluorescence

hiPSCs or hiPSC-CMs at 50%–70% confluency were fixed in 4% formaldehyde (Thermo Scientific, Thermo Fisher Scientific, Waltham, MA, USA) then stored in Dulbecco's phosphate buffered saline (DPBS; Gibco, Thermo Fisher Scientific, Waltham, MA, USA) containing 0.01% sodium azide at 4°C. Fixed cells were blocked and permeabilized in blocking buffer (5% normal goat serum and 0.3% Triton [Thermo Scientific, Thermo Fisher Scientific, Waltham, MA, USA] in DPBS). They were then washed in DPBS and incubated overnight at 4°C with primary antibody diluted in antibody dilution buffer (ADB; 1% BSA [Sigma-Aldrich, St. Louis, MO, USA]) and 0.3% Triton in DPBS). The following day cells were washed in DPBS and then incubated with secondary antibody in ADB for 1 h. Cells were then washed and SlowFade Diamond Antifade Mountant with DAPI (Thermo Fisher Scientific, Waltham, MA, USA) added. Cells were imaged using a Leica SP5 laser scanning confocal microscope (Leica Microsystems, Wetzlar, Germany). All antibodies used are described in Table S4.

hiPSC-CM RNA-seq

mRNA was extracted using the RNeasy Mini Kit as described previously and sequencing was performed by Novogene Europe (Cambridge, UK). For RNA-seq analysis, FASTQ files were quality-checked and processed using the nf-core/rnasplice pipeline (v.1.0.4).[45] Reads were aligned to the human reference genome GRCh38 (Ensembl release 114) using STAR (v.2.7.11b),[46] a splice-aware aligner. Transcript-level quantification was performed using Salmon (v.1.10.3).[47] Isoform usage was calculated as the proportion of total gene-level TPM contributed by each transcript within each sample using R (v.4.2.2).[48]

CRISPR-Cas9 gene-editing

A custom single guide RNA (gRNA) and homology-directed repair (HDR) template were designed and ordered from Integrated DNA Technologies (IDT, Coralville, IA, USA; sequences provided in Table S5). A ribonucleoprotein complex (RNP) was made by incubating 1.5 μL of Cas9 (IDT, Coralville, IA, USA) with 3 μL of gRNA. hiPSCs at 50% confluency were detached and suspended in 100 μL nucleofection buffer (P4 primary-cell 4D nucleofector kit, Lonza, Basel, Switzerland) with 3.5 μL RNP complex and 4 μL HDR template and nucleofected using the Lonza 4D-Nucleofector. Following nucleofection, hiPSCs were cultured with E8 Flex medium containing ROCK inhibitor and Alt-R HDR enhancer V2 (IDT, Coralville, IA, USA). Once nucleofected cells reached >80% confluency, they were detached and re-seeded to single colonies onto 60 mm plates. Once colonies were larger but not yet confluent with adjacent colonies, they were individually picked by pipette into a 96-well plate, then propagated, and then genotyped (described in the subsequent sections). Clones of the desired genotype were re-seeded onto 60 mm plate and the previous process repeated, picking 24 colonies. If all 24 wells were of the same genotype, this was considered satisfactory confirmation of the clone genotype and it was carried forward for experimentation.

Genotyping

For genotyping of CRISPR clones from 96-well plates, DNA was extracted using the Phire Tissue Direct PCR Master Mix kit. For all other genotyping (off targets, confirmation of genotype) DNA was extracted from pellets from a 6-well plate using a DNeasy Blood-and-Tissue kit (QIAGEN, Hilden, Germany), following the manufacturer's protocol. Extracted DNA was amplified by PCR using the Phire Tissue Direct PCR Master Mix Kit (Thermo Fisher Scientific, Waltham, MA, USA) and sequenced via Sanger sequencing performed by Source BioScience (Nottingham, UK). Primers used are provided in Table S2. All PCR primers used to confirm editing targeted sequences outside of the repair HDR template.

Assessment of off target mutations

Designed CRISPR gRNAs were assessed for predicted off target mutations using CRISPOR,[49] and predicted sites ranked by cutting frequency determination score. PCR primers were designed for the top five off target regions and genotyping was performed on all subclones. Primers used are provided in Table S2.

Assessment of karyotype abnormalities

Extracted hiPSC DNA was analyzed for common stem cell karyotype abnormalities using the hiPSC Genetic Analysis Kit (STEMCELL Technologies, Vancouver, British Columbia, Canada) following the manufacturer's instructions. Generated cell lines were compared to a company-provided control. Three replicates were run per genomic region per cell line, and the reaction was performed using the QuantStudio 5 Real-Time PCR system.

Titin protein measurement

Samples were prepared as described by Jiang et al.[50] In brief, snap-frozen pelleted hiPSC-CMs were placed in glass homogenisers with titin sample buffer (8 M urea [Sigma-Aldrich, St. Louis, MO, USA]), 2 M thiourea (Sigma-Aldrich, St. Louis, MO, USA), 3% SDS (Fisher Scientific, Thermo Fisher Scientific, Waltham, MA, USA), 0.03% bromophenol blue (Scientific Laboratory Supplies Ltd., Nottingham, UK), 75 mM dithiothreitol (VWR International, Radnor, PA, USA), 50 mM Tris-HCl pH 6.8 (Fisher Scientific, Thermo Fisher Scientific, Waltham, MA, USA) and 50% glycerol (Sigma-Aldrich, St. Louis, MO, USA) with complete EDTA-free protease-inhibitor tablets (Roche, Basel, Switzerland). Samples were homogenized using a glass pestle in a 60°C water bath for 30 s, then at room temperature for 2 min. They were placed on ice for 5 min then centrifuged for 5 min. The supernatant was aliquoted and immediately frozen. Samples were used after one thaw and not re-frozen.

SDS-polyacrylamide gels for titin quantification were prepared in-house. These were 15-well, 1.5 mm, two-phase gels comprising a 10% acrylamide lower third and a 2% acrylamide upper two-thirds. Gels were run at 4°C, at 3 mA per gel, in buffer comprising 25 mM Tris base (Sigma-Aldrich, St. Louis, MO, USA), 192 mM Glycine (Sigma-Aldrich, St. Louis, MO, USA), and 0.1% SDS. Gels were fixed in a 50% methanol (Sigma-Aldrich, St. Louis, MO, USA), 7% acetic acid (Fisher Scientific, Thermo Fisher Scientific, Waltham, MA, USA), solution then stained overnight in SYPRO Ruby stain (Invitrogen, Thermo Fisher Scientific, Waltham, MA, USA), and imaged the following day using a ChemiDoc MP Imaging System (Bio-Rad, Hercules, CA, USA). The upper phase of the gel was used for quantification of key titin isoforms while the whole lane of the lower phase captured the pool of other cardiac proteins ("other protein," OP) up to ∼250 kDa in size. Titin levels were normalized to OP from the same gel lane as per Fomin et al.[9] This normalization method was selected after comparison of other commonly used cardiomyocyte housekeepers (data not shown). All uncropped gels are shown in Figure S8.

Statistical analysis

Unless stated otherwise, all statistical analyses were performed using GraphPad Prism software v.10.0.3 (GraphPad Software Inc., San Diego, CA, USA).

For comparison between groups, normality was assessed using a Shapiro-Wilk test. Normally distributed data were analyzed using a two-sided unpaired Student's t test (two groups) or an ordinary one-way analysis of variance (ANOVA) with Tukey's multiple comparison test (more than two groups). Non-normally distributed data were analyzed using a Mann-Whitney test (two groups).

Copy number analysis of genomic regions frequently affected in hiPSC culture was performed using the analysis platform of STEMCELL Technologies, which applies a gradient-boosted machine learning algorithm to classify samples based on copy number and statistical significance (ANOVA). The platform classifies each region for each sample as either normal or having a putative or definitive chromosomal amplification or deletion.

DATA AND CODE AVAILABILITY

All data are included in the manuscript. Raw data are available upon request.

ACKNOWLEDGMENTS

R.B.K. is supported by the Rhodes Trust through a Rhodes Scholarship. This work was supported by the BHF's Big Beat Challenge award to CureHeart (awarded to H.W., BBC/F/21/220106). C.N.T. is supported by a Wellcome Sir Henry Dale Fellowship (222567/Z/21/Z). T.C.R. is funded as part of the MRC Centre of Research Excellence in Therapeutic Genomics.

AUTHOR CONTRIBUTIONS

R.B.K. and H.W. conceived the study; H.W., C.N.T., and A.J.S. supervised the work; R.B.K. performed the experimentation and analysis; M.M.-J. performed RNA-seq quality control and alignment; T.C.R. contributed methodology and materials for luciferase assay experiments; R.B.K. wrote the first draft of the manuscript. All authors contributed to the final manuscript.

DECLARATION OF INTERESTS

H.W. reports consultancies for Cytokinetics, BioMarin, Astra Zeneca, and CardiaTec, and is a founder and director of Cora Biosciences. A.J.S. acts as a consultant for Cora Biosciences but does not personally benefit from any compensation. T.C.R. is a founder and shareholder in Orfonyx Bio, a biotechnology company that aims to develop uORF-targeting therapeutics. T.C.R. is also a consultant for Orfonyx Bio and has filed multiple patents related to uORF targeting.

SUPPLEMENTAL INFORMATION

Supplemental information can be found online at https://doi.org/10.1016/j.omtn.2026.103077.

REFERENCES

1. Watkins, H., Ashrafian, H., and Redwood, C. (2011). Inherited cardiomyopathies. N. Engl. J. Med. *364*, 1643–1656. https://doi.org/10.1056/NEJMra0902923.
2. Hershberger, R.E., Hedges, D.J., and Morales, A. (2013). Dilated cardiomyopathy: the complexity of a diverse genetic architecture. Nat. Rev. Cardiol. *10*, 531–547. https://doi.org/10.1038/nrcardio.2013.105.
3. Fatkin, D., Huttner, I.G., Kovacic, J.C., Seidman, J.G., and Seidman, C.E. (2019). Precision medicine in the management of dilated cardiomyopathy: JACC state-of-the-art review. J. Am. Coll. Cardiol. *74*, 2921–2938. https://doi.org/10.1016/j.jacc.2019.10.011.
4. Huggins, G.S., Kinnamon, D.D., Haas, G.J., Jordan, E., Hofmeyer, M., Kransdorf, E., Ewald, G.A., Morris, A.A., Owens, A., Lowes, B., et al. (2022). Prevalence and cumulative risk of familial idiopathic dilated cardiomyopathy. JAMA *327*, 454–463. https://doi.org/10.1001/jama.2021.24674.
5. Herman, D.S., Lam, L., Taylor, M.R.G., Wang, L., Teekakirikul, P., Christodoulou, D., Conner, L., DePalma, S.R., McDonough, B., Sparks, E., et al. (2012). Truncations of titin causing dilated cardiomyopathy. N. Engl. J. Med. *366*, 619–628. https://doi.org/10.1056/NEJMoa1110186.
6. Loescher, C.M., Hobbach, A.J., and Linke, W.A. (2022). Titin (*TTN*): from molecule to modifications, mechanics, and medical significance. Cardiovasc. Res. *118*, 2903–2918. https://doi.org/10.1093/cvr/cvab328.
7. Wang, S., Zhang, Z., He, J., Liu, J., Guo, X., Chu, H., Xu, H., and Wang, Y. (2023). Comprehensive review on gene mutations contributing to dilated cardiomyopathy. Front. Cardiovasc. Med. *10*, 1296389. https://doi.org/10.3389/fcvm.2023.1296389.
8. McAfee, Q., Chen, C.Y., Yang, Y., Caporizzo, M.A., Morley, M., Babu, A., Jeong, S., Brandimarto, J., Bedi, K.C., Jr., Flam, E., et al. (2021). Truncated titin proteins in dilated cardiomyopathy. Sci. Transl. Med. *13*, eabd7287. https://doi.org/10.1126/scitranslmed.abd7287.
9. Fomin, A., Gärtner, A., Cyganek, L., Tiburcy, M., Tuleta, I., Wellers, L., Folsche, L., Hobbach, A.J., von Frieling-Salewsky, M., Unger, A., et al. (2021). Truncated titin proteins and titin haploinsufficiency are targets for functional recovery in human cardiomyopathy due to *TTN* mutations. Sci. Transl. Med. *13*, eabd3079. https://doi.org/10.1126/scitranslmed.abd3079.
10. Ghahremani, S., Kanwal, A., Pettinato, A., Ladha, F., Legere, N., Thakar, K., Zhu, Y., Tjong, H., Wilderman, A., Stump, W.T., et al. (2024). CRISPR Activation Reverses Haploinsufficiency and Functional Deficits Caused by TTN Truncation Variants. Circulation *149*, 1285–1297. https://doi.org/10.1161/CIRCULATIONAHA.123.063972.
11. Desai, M.Y., Nagueh, S.F., Giudicessi, J.R., Previs, M.J., Kellner, D., Pollman, M.J., Varfaj, F., Robertson, L.A., Sonicheva-Paterson, N., Pushkin, R., et al. (2025). First-in-human study of TN-201, an AAV9 gene replacement therapy in MYBPC3-associated hypertrophic cardiomyopathy: initial safety, pharmacodynamic, and imaging results from MyPEAK-1. Cardiovasc. Res. *121*, 2628–2631. https://doi.org/10.1093/cvr/cvaf200.
12. Greenberg, B., Taylor, M., Adler, E., Colan, S., Ricks, D., Yarabe, P., Battiprolu, P., Shah, G., Patel, K., Coggins, M., et al. (2025). Phase 1 study of AAV9. LAMP2B gene therapy in Danon disease. N. Engl. J. Med. *392*, 972–983. https://doi.org/10.1056/NEJMoa2412392.
13. Calvo, S.E., Pagliarini, D.J., and Mootha, V.K. (2009). Upstream open reading frames cause widespread reduction of protein expression and are polymorphic among humans. Proc. Natl. Acad. Sci. USA *106*, 7507–7512. https://doi.org/10.1073/pnas.0810916106.
14. Hanson, B., Svrzikapa, N., Feng, N., Friedrichsen, H.J., Lennaárd, A.J., Abuhamdah, R., Hemmer, N., Chwalenia, K., Drake, M., Jad, Y., et al. (2025). Toward a model of uORF-mediated translational control: An integrated bioinformatic and experimental approach. Preprint at bioRxiv. https://doi.org/10.1101/2025.07.28.667205.
15. Liang, X.-H., Shen, W., Sun, H., Migawa, M.T., Vickers, T.A., and Crooke, S.T. (2016). Translation efficiency of mRNAs is increased by antisense oligonucleotides targeting upstream open reading frames. Nat. Biotechnol. *34*, 875–880. https://doi.org/10.1038/nbt.3589.
16. Ahlskog, N., Svrzikapa, N., Abuhamdah, R., Kye, M., Jad, Y., Feng, N., Hanson, B., Wood, M.J.A., and Roberts, T.C. (2025). uORF-targeting steric block antisense oligonucleotides do not reproducibly increase RNASEH1 expression. Mol. Ther. Nucleic Acids *36*, 102406. https://doi.org/10.1016/j.omtn.2024.102406.
17. Doisy, M., Gaci, A., Soukarieh, O., Proust, C., Trégouët, D.-A., and Goyenvalle, A. (2026). Targeting a Pathogenic Variant Creating an Upstream AUG in the ENG 5′ Untranslated Region with Antisense Oligonucleotides Fails to Restore Protein Expression. Nucleic Acid Therapeut. *36*, 12–22. https://doi.org/10.1177/21593337251396711.
18. Hedaya, O.M., Venkata Subbaiah, K.C., Jiang, F., Xie, L.H., Wu, J., Khor, E.-S., Zhu, M., Mathews, D.H., Proschel, C., and Yao, P. (2023). Secondary structures that regulate mRNA translation provide insights for ASO-mediated modulation of cardiac hypertrophy. Nat. Commun. *14*, 6166. https://doi.org/10.1038/s41467-023-41799-1.
19. Ang, Z., Paruzzo, L., Hayer, K.E., Schmidt, C., Torres Diz, M., Xu, F., Zankharia, U., Zhang, Y., Soldan, S., Zheng, S., et al. (2023). Alternative splicing of its 5′-UTR limits CD20 mRNA translation and enables resistance to CD20-directed immunotherapies. Blood *142*, 1724–1739. https://doi.org/10.1182/blood.2023020400.
20. de Breyne, S., Polvèche, H., Auboeuf, D., Ohlmann, T., Gruffat, H., and Manet, E. (2025). Alternative splicing in the RBMXL1 5′-UTR induces uORF-mediated translation control in activated B lymphocytes. Sci. Rep. *15*, 22800. https://doi.org/10.1038/s41598-025-03274-3.
21. Feng, N., Goedert, T., Svrzikapa, N., Yan, D., Friedrichsen, H.J., Hanson, B., Ljungdahl, A., Dafinca, R., Talbot, K., Sanders, S.J., et al. (2026). Targeted BDNF upregulation via upstream open reading frame disruption. Mol. Ther. *34*, 1652–1671. https://doi.org/10.1016/j.ymthe.2025.12.024.
22. Yan, H., and Tang, W. (2024). Programmed RNA editing with an evolved bacterial adenosine deaminase. Nat. Chem. Biol. *20*, 1361–1370. https://doi.org/10.1038/s41589-024-01661-x.
23. Huey, D.J., Guadarrama, E., DeKeyser, J.-M., Vanoye, C.G., Simmons, C.Q., Li, Q., Stroup, E.K., Ji, Z., and George, A.L., Jr. (2026). An upstream open reading frame represses translation of the neuronal potassium channel KCNQ2. Proc. Natl. Acad. Sci. USA *123*, e2610782123. https://doi.org/10.1073/pnas.2610782123.
24. Cadar, A.G., Zhong, L., Lin, A., Valenzuela, M.O., and Lim, C.C. (2014). Upstream open reading frame in 5′-untranslated region reduces titin mRNA translational efficiency. Biochem. Biophys. Res. Commun. *453*, 185–191. https://doi.org/10.1016/j.bbrc.2014.09.085.

25. Filatova, A., Reveguk, I., Piatkova, M., Bessonova, D., Kuziakova, O., Demakova, V., Romanishin, A., Fishman, V., Imanmalik, Y., Chekanov, N., et al. (2023). Annotation of uORFs in the OMIM genes allows to reveal pathogenic variants in 5′ UTRs. Nucleic Acids Res. *51*, 1229–1244. https://doi.org/10.1093/nar/gkac1247.

26. Roberts, A.M., Ware, J.S., Herman, D.S., Schafer, S., Baksi, J., Bick, A.G., Buchan, R.J., Walsh, R., John, S., Wilkinson, S., et al. (2015). Integrated allelic, transcriptional, and phenomic dissection of the cardiac effects of titin truncations in health and disease. Sci. Transl. Med. *7*, 270ra6. https://doi.org/10.1126/scitranslmed.3010134.

27. Michel, A.M., Kiniry, S.J., O'Connor, P.B.F., Mullan, J.P., and Baranov, P.V. (2018). GWIPS-viz: 2018 update. Nucleic Acids Res. *46*, D823–D830. https://doi.org/10.1093/nar/gkx790.

28. Kiniry, S.J., Michel, A.M., and Baranov, P.V. (2018). The GWIPS-viz browser. Curr. Protoc. Bioinform. *62*, e50. https://doi.org/10.1002/cpbi.50.

29. Kearse, M.G., and Wilusz, J.E. (2017). Non-AUG translation: a new start for protein synthesis in eukaryotes. Genes Dev. *31*, 1717–1731. https://doi.org/10.1101/gad.305250.117.

30. de Villiers, C., Ormondroyd, E., Thomson, K., Ormerod, J.O.M., Sarwar, R., Waring, A., Bagnall, R.D., Sparrow, A., Steeples, V., Blair, E., et al. (2026). Hypertrophic cardiomyopathy caused by Filamin-C (FLNC) variants has restrictive and extracardiac features and a distinctive ECG. Heart Rhythm *23*, 1289–1299. https://doi.org/10.1016/j.hrthm.2026.01.054.

31. Simkin, D., Papakis, V., Bustos, B.I., Ambrosi, C.M., Ryan, S.J., Baru, V., Williams, L.A., Dempsey, G.T., McManus, O.B., Landers, J.E., et al. (2022). Homozygous might be hemizygous: CRISPR/Cas9 editing in iPSCs results in detrimental on-target defects that escape standard quality controls. Stem Cell Rep. *17*, 993–1008. https://doi.org/10.1016/j.stemcr.2022.02.008.

32. Lorenz, R., Bernhart, S.H., Höner zu Siederdissen, C., Tafer, H., Flamm, C., Stadler, P.F., and Hofacker, I.L. (2011). ViennaRNA Package 2.0. Algorithm Mol. Biol. *6*, 26. https://doi.org/10.1186/1748-7188-6-26.

33. Guarracino, A., Pepe, G., Ballesio, F., Adinolfi, M., Pietrosanto, M., Sangiovanni, E., Vitale, I., Ausiello, G., and Helmer-Citterich, M. (2021). BRIO: a web server for RNA sequence and structure motif scan. Nucleic Acids Res. *49*, W67–W71. https://doi.org/10.1093/nar/gkab400.

34. Desmet, F.-O., Hamroun, D., Lalande, M., Collod-Béroud, G., Claustres, M., and Béroud, C. (2009). Human Splicing Finder: an online bioinformatics tool to predict splicing signals. Nucleic Acids Res. *37*, e67. https://doi.org/10.1093/nar/gkp215.

35. Ly, J., Xiang, K., Su, K.-C., Sissoko, G.B., Bartel, D.P., and Cheeseman, I.M. (2024). Nuclear release of eIF1 restricts start-codon selection during mitosis. Nature *635*, 490–498. https://doi.org/10.1038/s41586-024-08088-3.

36. Gramlich, M., Pane, L.S., Zhou, Q., Chen, Z., Murgia, M., Schötterl, S., Goedel, A., Metzger, K., Brade, T., Parrotta, E., et al. (2015). Antisense-mediated exon skipping: a therapeutic strategy for titin-based dilated cardiomyopathy. EMBO Mol. Med. *7*, 562–576. https://doi.org/10.15252/emmm.201505047.

37. Celik, S., Hyrefelt, L., Czuba, T., Li, Y., Assis, J., Martinez, J., Johansson, M., André, O., Synnergren, J., Sandstedt, J., et al. (2025). Antisense-mediated regulation of exon usage in the elastic spring region of Titin modulates sarcomere function. Cardiovasc. Res. *121*, 629–642. https://doi.org/10.1093/cvr/cvaf037.

38. Sasaki, S., Sun, R., Bui, H.-H., Crosby, J.R., Monia, B.P., and Guo, S. (2019). Steric inhibition of 5′ UTR regulatory elements results in upregulation of human CFTR. Mol. Ther. *27*, 1749–1757. https://doi.org/10.1016/j.ymthe.2019.06.016.

39. Kidwell, A., Yadav, S.P.S., Maier, B., Zollman, A., Ni, K., Halim, A., Janosevic, D., Myslinski, J., Syed, F., Zeng, L., et al. (2023). Translation rescue by targeting Ppp1r15a through its upstream open reading frame in sepsis-induced acute kidney injury in a murine model. J. Am. Soc. Nephrol. *34*, 220–240. https://doi.org/10.1681/ASN.2022060644.

40. Liang, X.-H., Sun, H., Shen, W., Wang, S., Yao, J., Migawa, M.T., Bui, H.-H., Damle, S.S., Riney, S., Graham, M.J., et al. (2017). Antisense oligonucleotides targeting translation inhibitory elements in 5′ UTRs can selectively increase protein levels. Nucleic Acids Res. *45*, 9528–9546. https://doi.org/10.1093/nar/gkx632.

41. Chu, Y., Yu, D., Li, Y., Huang, K., Shen, Y., Cong, L., Zhang, J., and Wang, M. (2024). A 5′ UTR language model for decoding untranslated regions of mRNA and function predictions. Nat. Mach. Intell. *6*, 449–460. https://doi.org/10.1038/s42256-024-00823-9.

42. Manske, F., Ogoniak, L., Jürgens, L., Grundmann, N., Makałowski, W., and Wethmar, K. (2023). The new uORFdb: integrating literature, sequence, and variation data in a central hub for uORF research. Nucleic Acids Res. *51*, D328–D336. https://doi.org/10.1093/nar/gkac899.

43. Liu, Q., Peng, X., Shen, M., Qian, Q., Xing, J., Li, C., and Gregory, R.I. (2023). Ribo-uORF: a comprehensive data resource of upstream open reading frames (uORFs) based on ribosome profiling. Nucleic Acids Res. *51*, D248–D261. https://doi.org/10.1093/nar/gkac1094.

44. Dyer, S.C., Austine-Orimoloye, O., Azov, A.G., Barba, M., Barnes, I., Barrera-Enriquez, V.P., Becker, A., Bennett, R., Beracochea, M., Berry, A., et al. (2025). Ensembl 2025. Nucleic Acids Res. *53*, D948–D957. https://doi.org/10.1093/nar/gkae1071.

45. Ashmore, J. (2024). nf-core/rnasplice (Version 1.0.4). nf-core. https://doi.org/10.5281/zenodo.8424632.

46. Dobin, A., Davis, C.A., Schlesinger, F., Drenkow, J., Zaleski, C., Jha, S., Batut, P., Chaisson, M., and Gingeras, T.R. (2013). STAR: ultrafast universal RNA-seq aligner. Bioinformatics *29*, 15–21. https://doi.org/10.1093/bioinformatics/bts635.

47. Patro, R., Duggal, G., Love, M.I., Irizarry, R.A., and Kingsford, C. (2017). Salmon provides fast and bias-aware quantification of transcript expression. Nat. Methods *14*, 417–419. https://doi.org/10.1038/nmeth.4197.

48. R Core Team (2021). R: A Language and Environment for Statistical Computing (R Foundation for Statistical Computing). https://www.R-project.org/.

49. Concordet, J.-P., and Haeussler, M. (2018). CRISPOR: intuitive guide selection for CRISPR/Cas9 genome editing experiments and screens. Nucleic Acids Res. *46*, W242–W245. https://doi.org/10.1093/nar/gky354.

50. Jiang, H., Hooper, C., Kelly, M., Steeples, V., Simon, J.N., Beglov, J., Azad, A.J., Leinhos, L., Bennett, P., Ehler, E., et al. (2021). Functional analysis of a gene-edited mouse model to gain insights into the disease mechanisms of a titin missense variant. Basic Res. Cardiol. *116*, 14–18. https://doi.org/10.1007/s00395-021-00853-z.

OMTN, Volume *37*

Supplemental information

Functional analysis of *TTN* uORFs reveals context-dependent translational regulation

Rosemary B. Kirk, Alexander J. Sparrow, Marta Moya-Jódar, Thomas C. Roberts, Christopher N. Toepfer, and Hugh Watkins

Supplemental material

Table S1: Engineered hiPSC lines do not show predicted off-target mutations.

The guide RNA used for engineering of hiPSC lines was assessed for predicted off-target mutations using CRISPOR. PCR primers were designed for the top five off-target regions and genotyping performed on all subclones.

Loci (hg19)	Off target score	Mismatch	Genomic location	Result<br>WT	 WT-ΔuORF	 *TTN*$^{+/-}$	 *TTN*$^{+/-}$-ΔuORF
Chr2:215157979-215158001	0.535	AGTGGAACACCAA TTCATGT(TGG)	Intergenic	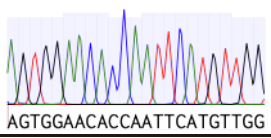	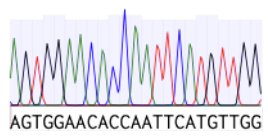	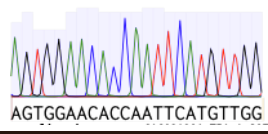	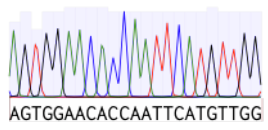
Chr5:17271161-17271183	0.533	ACAGGCTTACCG ATTAATAT(TGG)	Intergenic	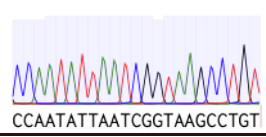	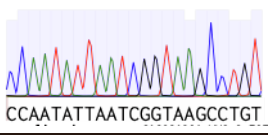	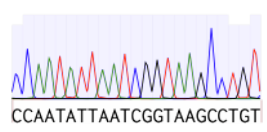	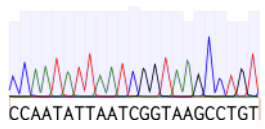
Chr3:172667593-172667615	0.528	AGAGACTCATCA ATTCATGA(GGG)	Intron	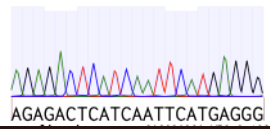	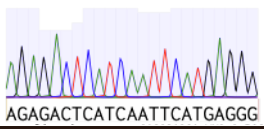	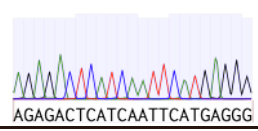	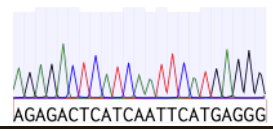
Chr11:20088531-20088553	0.369	GCAGGCTCCCCA ATTCATGT(GGG)	Intron	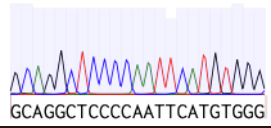	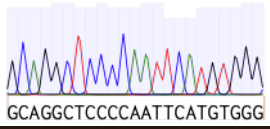	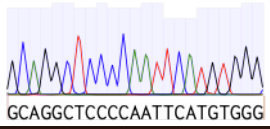	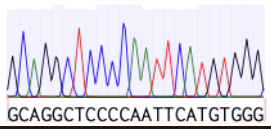
Chr18:66515999-66516021	0.320	AGAGGATCAAAC ATTCATGT(AGG)	Intergenic	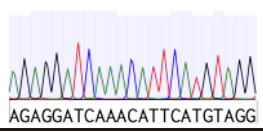	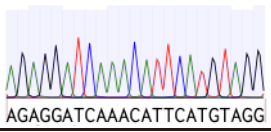	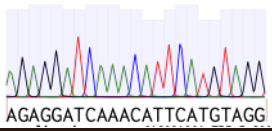	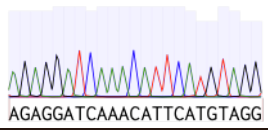

Table S2: Primers used for genotyping and RT-qPCR.

Target gene	Application	Sequence (5'-3')	Notes
Renilla luciferase	RT-qPCR	F: GTAACGCTGCCTCCAGCTAC<br>R: CCAAGCGGTGAGGTACTTGT	Quantification of mRNA levels of *Renilla* luciferase from dual reporter assays (under 5'-UTR control).
Firefly luciferase	RT-qPCR	F: ACTCTAAGACCGACTACCAGG<br>R: GTAGACCCAGAGCTGTTCATG	Quantification of mRNA levels of Firefly luciferase from dual reporter assays (internal control).
TTN	Genotyping	F: TAGGTCCTCTCCCATCATGCTT<br>R: ACAGGCAAAGAGAGTCTAGAAAAGA	For PCR and sequencing of *TTN* uORF region to genotype hiPSC lines.
TTN	Genotyping	F: CTCCCCAATATTTTTCGTTCACTCT<br>F_int1: TGCCTATCAGTTTCAGAGACATT<br>F_int2: GATGAACAACTTTCTGTGTCTAACT<br>F_int3: GGGGCTGAAGGTATGCGTCT<br>F_int4: GGTCAGCACTGGCAAGAAATAG<br>R_int3: CGCCTCGGGTGTAGGGATT<br>R_int2: CTTCTCTGAGCCTCAACACAGT<br>R_int1: CTGTCCCCGAAATACAAGTGGA<br>R: CACCTCCTATCCACTTCCCATT	For PCR and sequencing of *TTN* uORF region inclusive of flanking SNVs. F and R used for PCR amplification and sequencing, internal (“int”) primers used for further sequencing to confirm no sequence drop-off within the product.
Off-target 1	Genotyping	F: CATGCCTTACCTTGAGCCGTA<br>R: GGATTACCAGACCAGCTCACC	
Off-target 2	Genotyping	F: TGTGTGGAACCTGTGTGCTAA<br>R: AAGCTAATTCTGGAGCTGTCCTT	
Off-target 3	Genotyping	F: TCTTCAGGGATTTTAATGCTTGGC<br>R: GAGTTGAGGTAGGGTAGGCA	
Off-target 4	Genotyping	F: GTGAACTAATGGAGATGCTGACA<br>R: AATGTGGCCTTCATCTTCGTG	
Off-target 5	Genotyping	F: AATGCATTTGAAAGTCTGGTGTGC<br>R: ACAATGCCTGTGTCTCTGAAAGTA	

Table S3: TaqMan probes used for RT-qPCR.

Target gene	Probe ID	Supplier	Reporter dye
TTN	Hs00399225_m1	Thermo Fisher Scientific	FAM
ACTN2	Hs00153809_m1	Thermo Fisher Scientific	VIC

Table S4: Antibodies used for immunofluorescence.

Target protein	Host species	Supplier	Catalogue no.	Dilution	Application
Nanog (D73G4)	Rabbit	Cell Signaling Technology	#99399	1:100	IF
Oct4 (C30A3)	Rabbit	Cell Signaling Technology	#94310	1:100	IF
Sox2 (D6D9)	Rabbit	Cell Signaling Technology	#34516	1:100	IF
Titin (M-line)	Rabbit	Myomedix	#TTN-9	1:100	IF
α-actinin (EA53)	Mouse	Sigma-Aldrich	#A7732	1:400	IF
Secondary (anti-rabbit Alexa Fluo 488)	Goat	Invitrogen	#A11008	1:200	IF
Secondary (anti-mouse Alexa Fluo 568)	Goat	Invitrogen	#A11019	1:200	IF

Table S5: guide RNA and HDR sequence for ΔuORF CRISPR-Cas9 gene-editing.

Component	Sequence
Guide	AGAGGCTCACCGATTCATGT
HDR	GCTCACCTGATTTCTCAAGAGTGCCTAAAAAGGGTGGGACTAAGCCCAAGGTTGCTTCTGAAACGACG TCCTATGGAGAGTTGGTTTTTCTGACCTTCTCCGACTTGAATCGGTGAGCCTCTAATCCTAAAAACAAAA CTACACAGTCAAGACTGTGTAGTTTTGCTTTTCTATGCAATCCCTACACCCGAGGCGAGGCT

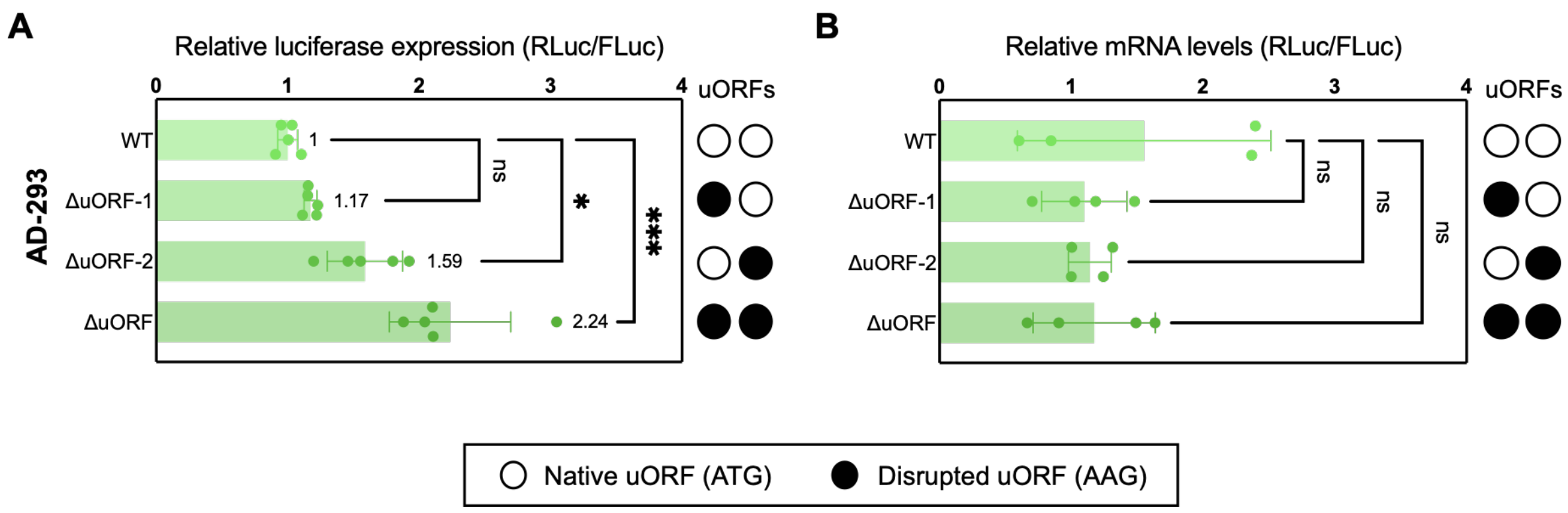

Figure S1: Individual disruption of *TTN* uORFs in a dual luciferase reporter assay in AD-293 cells.

(A) AD-293 cells were transfected with *TTN* 5' UTR dual luciferase reporter constructs containing the *TTN* 5' UTR in wild-type form (WT), with uORF-1 disrupted (ΔuORF-1), uORF-2 disrupted (ΔuORF-2), or both uORFs disrupted (ΔuORF). Luciferase activity was measured 24 hours post-transfection.

(B) AD-293 cells were transfected as in (A) and harvested 24 hours post-transfection for analysis of *Renilla* and firefly luciferase mRNA levels by RT-qPCR.

Bars represent mean (SD); $n = 5$ biological replicates each containing 4 technical replicates for (A) and $n = 4$ biological replicates each containing 2 technical replicates for (B). Values were normalised such that the mean of the WT control group was returned to a value of 1. Statistical significance was determined by performing a Kruskal-Wallis test with Dunnett's multiple comparisons test. *$p < 0.05$, ***$p < 0.001$; ns, not significant.

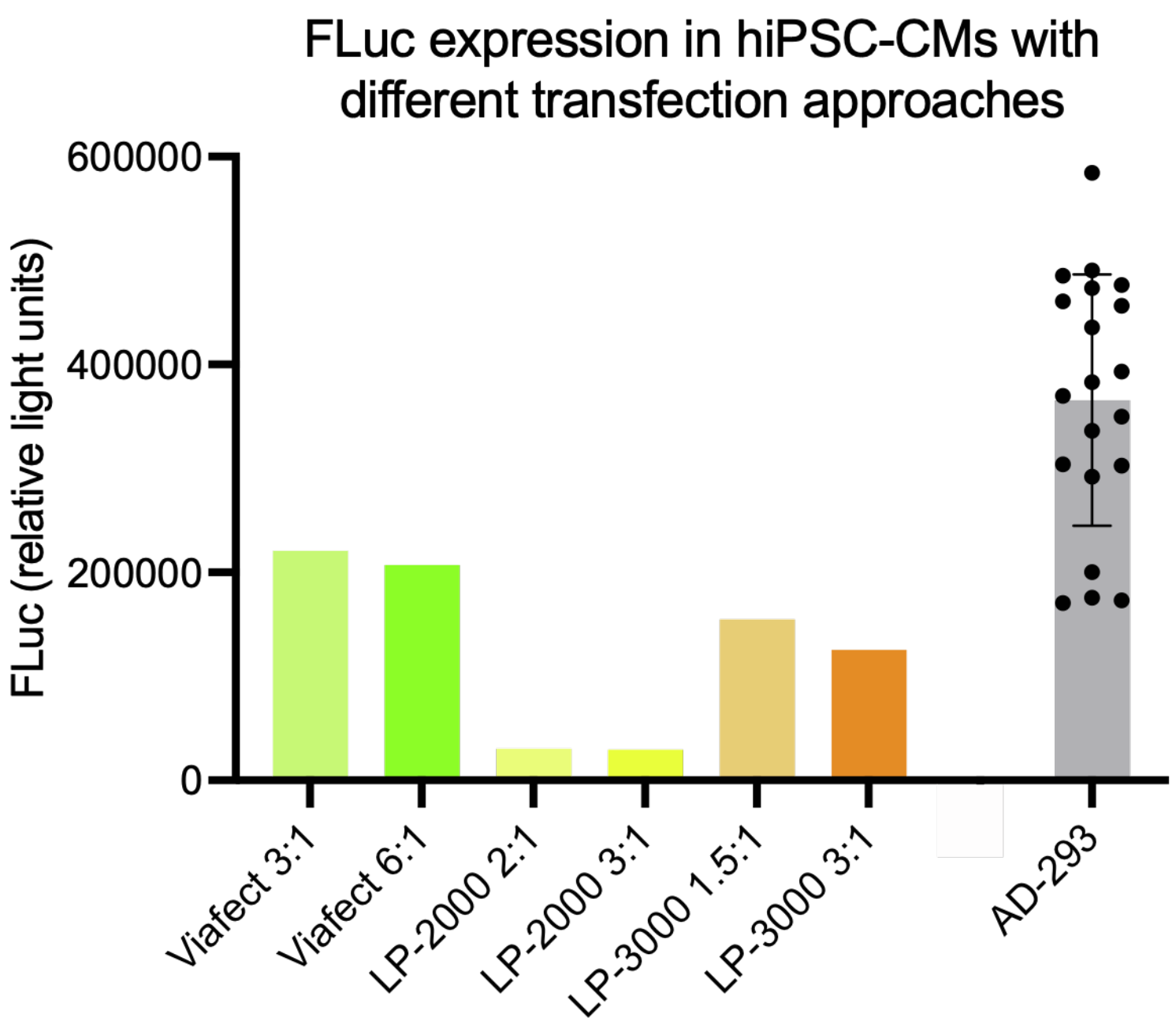

Figure S2: Optimisation of transfection conditions for dual luciferase reporter assays in hiPSC-derived cardiomyocytes.

Raw firefly luciferase activity measured 24 hours after transfection of hiPSC-CMs with the dual luciferase reporter plasmid using ViaFect, Lipofectamine 2000 (LP-2000), or Lipofectamine 3000 (LP-3000) at two reagent:DNA ratios (n = 1 per condition). Raw firefly luciferase activity from AD-293 cells transfected with the same reporter construct under identical assay conditions is shown for comparison (n = 20; 4 technical replicates from 5 independent experiments).

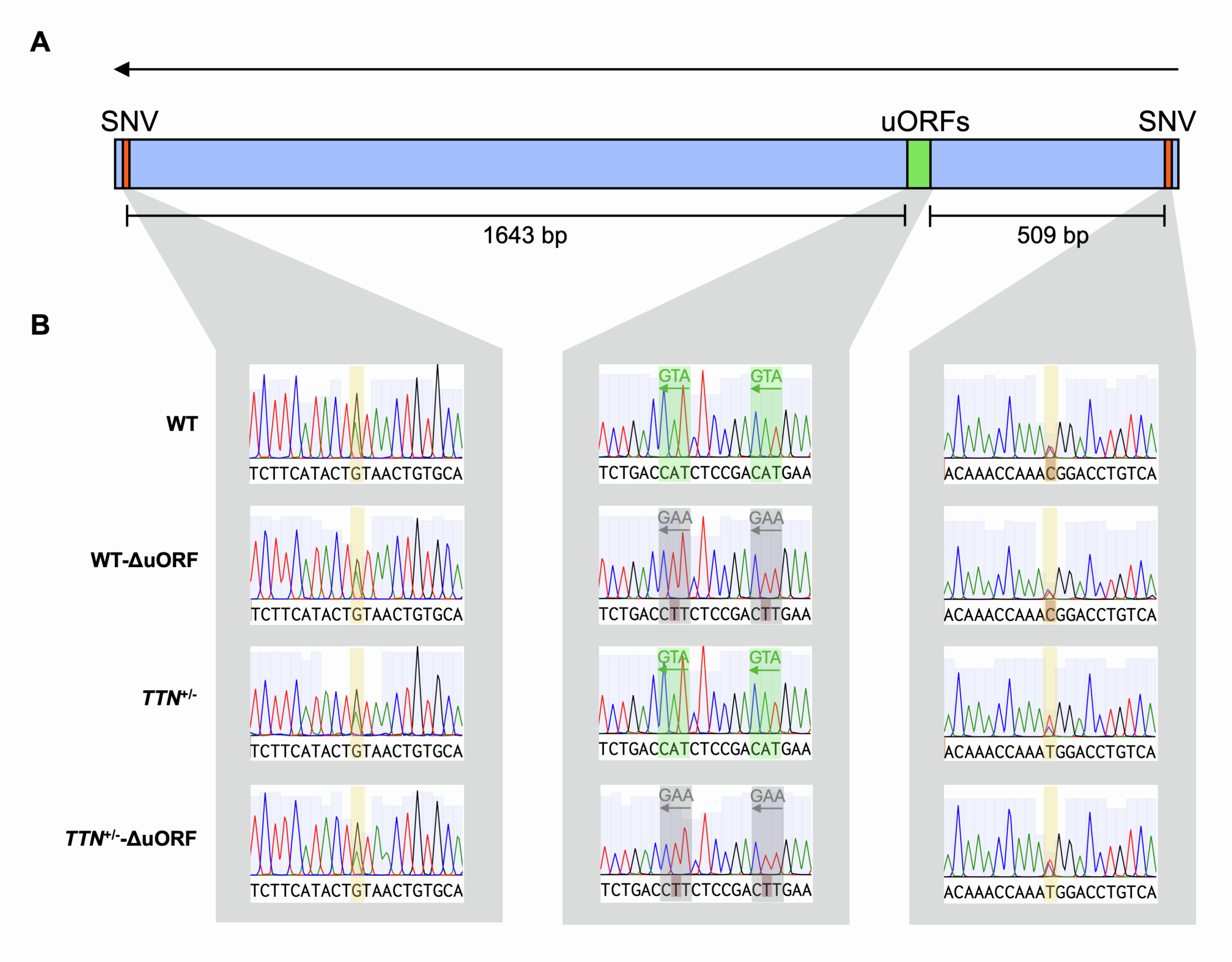

Figure S3: Engineered hiPSC lines show successful introduction of homozygous uORF disruption without evidence of large indels.

To exclude the possibility that apparently homozygous sequencing traces reflected monoallelic editing with a large indel in the second allele, the closest upstream and downstream heterozygous SNVs (**A**) were sequenced to confirm the presence of both alleles. Representative sequencing traces of the SNVs and the edit site are shown for all generated cell lines (**B**). Heterozygous SNVs are highlighted in yellow, uORF start codons in green, and disrupted uORF start codons in grey. As *TTN* is encoded on the reverse strand, the complementary sequence of the uORF start codons is annotated.

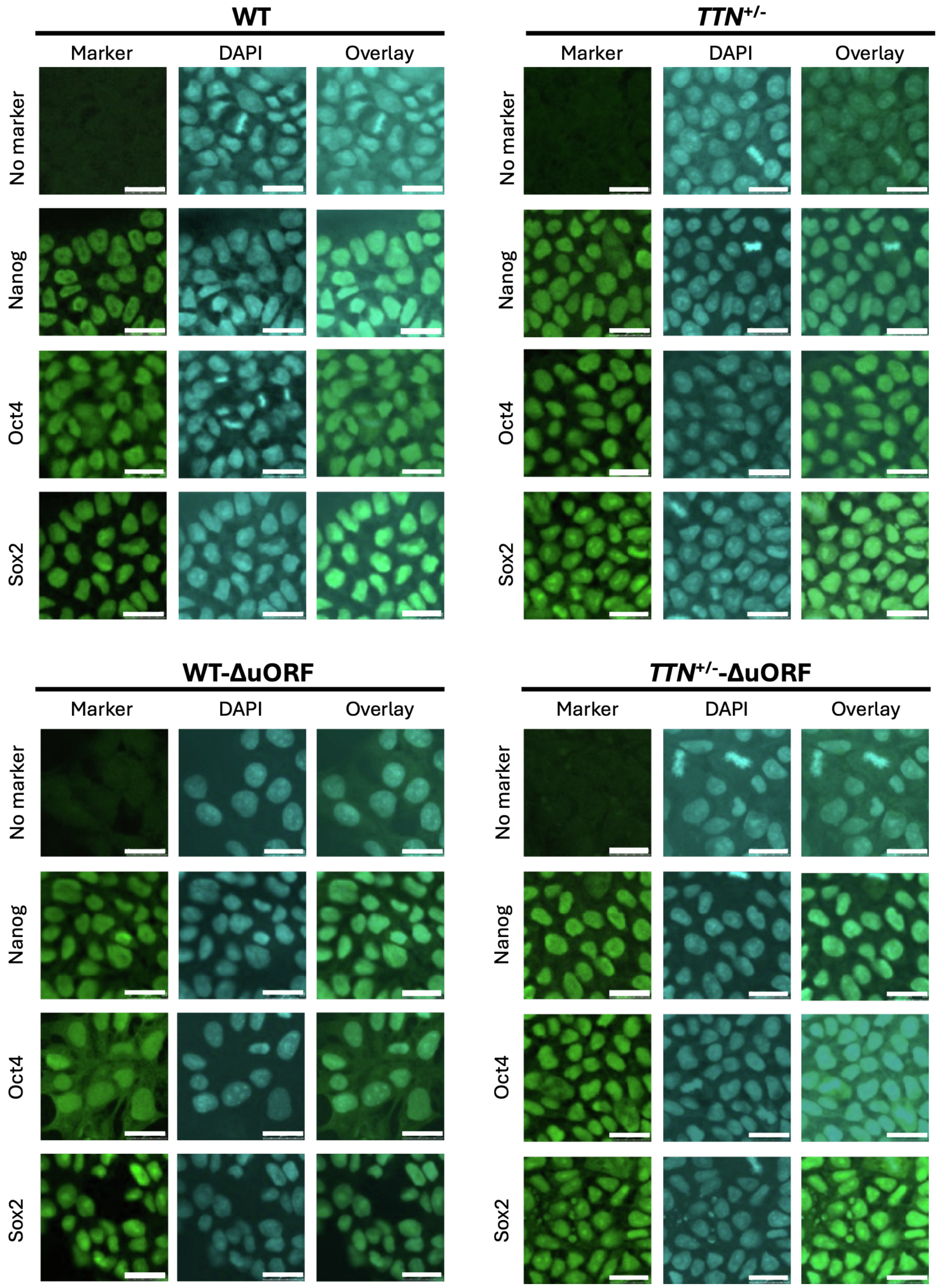

Figure S4: Engineered hiPSC lines express key pluripotency markers.

hiPSCs from all four generated cell lines (WT, WT-ΔuORF, *TTN*$^{+/-}$, *TTN*$^{+/-}$-ΔuORF) were fixed with 4% PFA/DPBS and incubated with rabbit IgG monoclonal antibodies targeting pluripotency markers Nanog, Oct4, and Sox2. All were incubated with secondary antibody and with DAPI as a nuclear marker, and imaged by confocal microscopy. Images show co-localisation of each pluripotency marker (green) with nuclear DAPI-stained DNA (blue). Images acquired with a 63× oil immersion objective with 3× magnification. Scale bar (white) 20 μm.

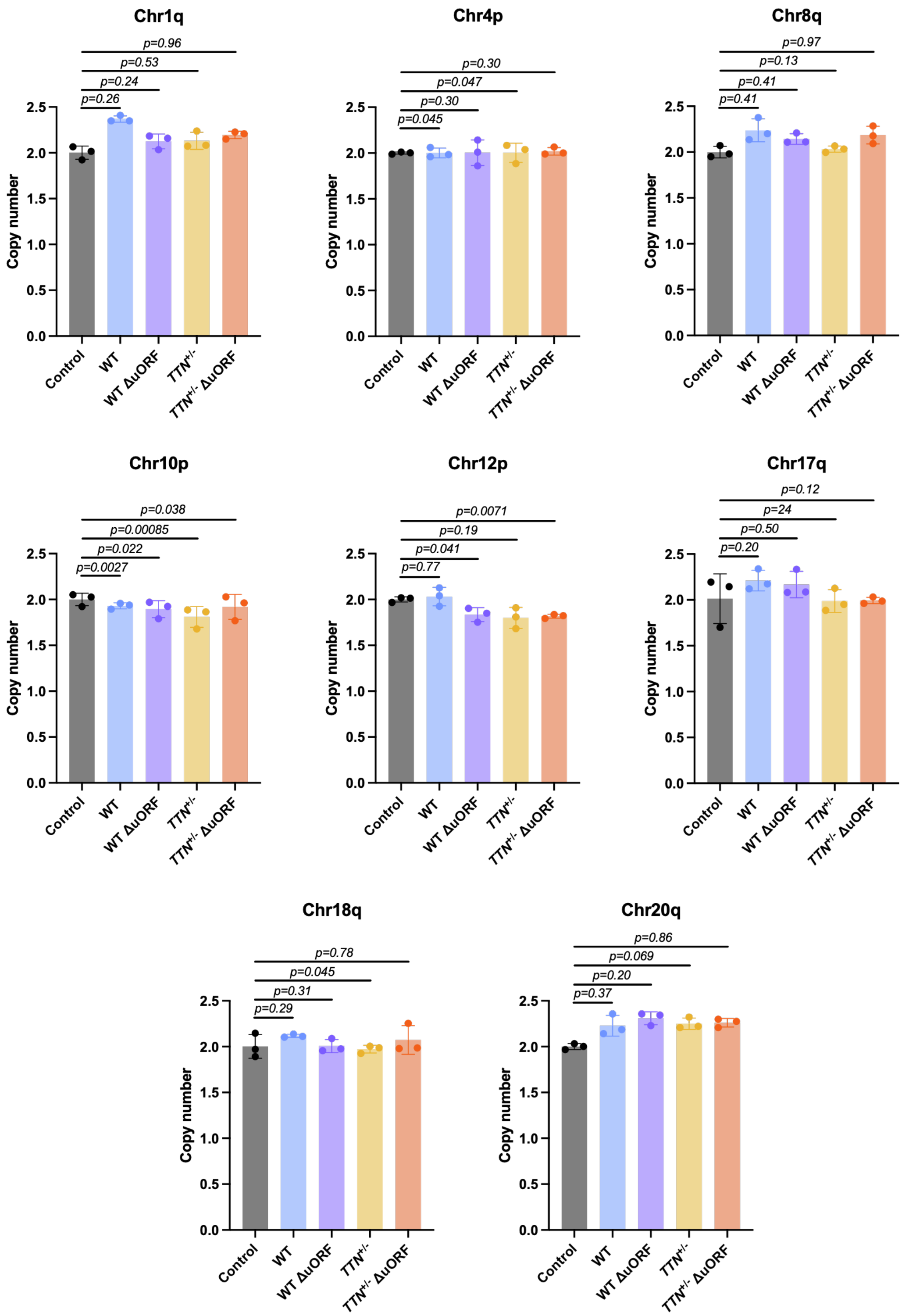

Figure S5: Engineered hiPSC lines do not have common hiPSC karyotype abnormalities.

Copy number analysis of genomic regions frequently affected in hiPSC culture was performed for each engineered hiPSC line (WT, WT-ΔuORF, *TTN*$^{+/-}$, *TTN*$^{+/-}$-ΔuORF) compared to a company-provided control using RT-qPCR. Bars represent mean (SD); n = 3 technical replicates. Data were analysed using the STEMCELL Technologies analysis platform, which applies a gradient-boosted machine learning algorithm to classify samples based on copy number and statistical significance (ANOVA). p-values are shown. No samples were classified as having putative or definitive chromosomal amplifications or deletions.

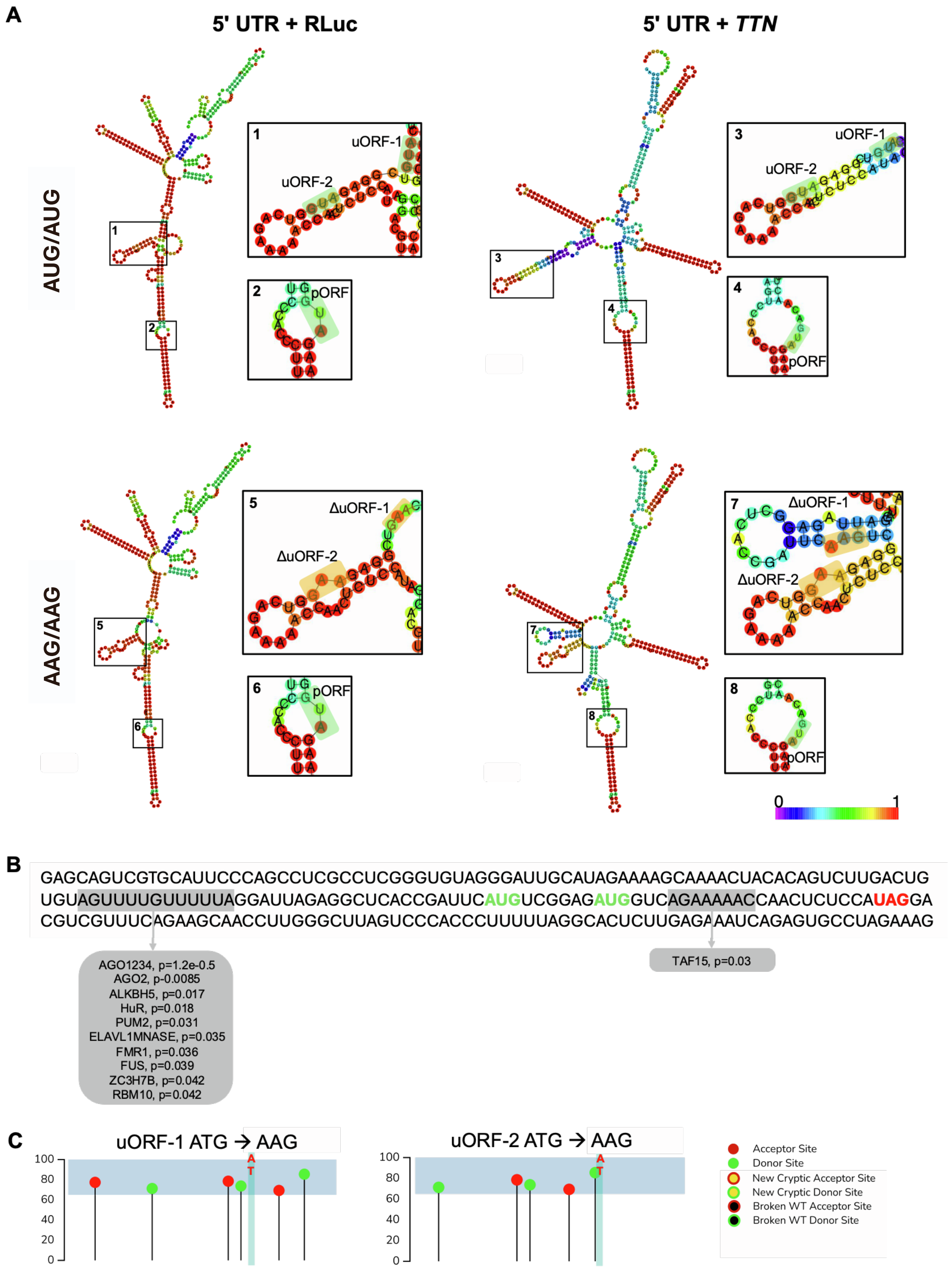

Figure S6: ***In silico*** **analyses do not predict that altered sequence context or uORF disruption alter gene expression via alternative mechanisms.**

(A) RNA secondary structure predictions generated using RNAfold for the *TTN* 5' UTR followed by either *Renilla* luciferase or *TTN* sequence (400 bp total), with wild-type uORF sequence (AUG/AUG) or uORF disruption (AAG/AAG). No major changes in predicted secondary structures were observed.

(B) Analysis of RNA-binding motifs within the *TTN* 5' UTR using BRIO (BEAM RNA Interaction mOtifs) shows several motifs in the 5' UTR, but none that would be disrupted by mutagenesis of the uORF start codons.

(C) Human Splicing Finder analysis predicting the effects of the nucleotide substitutions used to disrupt uORF-1 and uORF-2 (T → A) on splice donor and acceptor sites. Neither substitution is predicted to alter splicing sites.

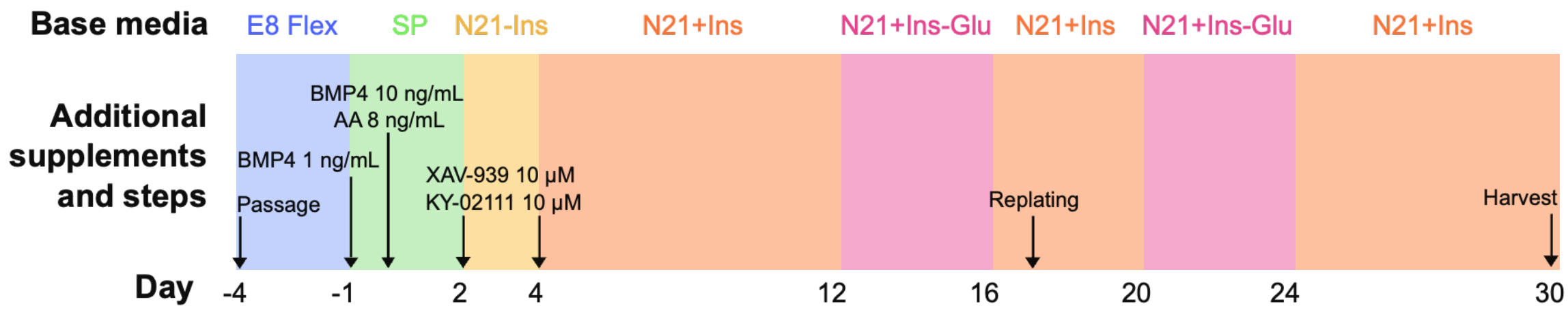

Figure S7: hiPSC-CM differentiation protocol.

hiPSCs were differentiated into hiPSC-CMs following a 30-day protocol. hiPSCs were passaged and maintained in E8 Flex, then transitioned through StemPro34 (SP), RPMI with N21-MAX Insulin Free Media Supplement (N21-Ins), RPMI with N21-MAX Media Supplement (N21+Ins), and RPMI medium with no glucose with N21-MAX Media Supplement (N21+Ins-Glu). Additional supplements were added including Bone Morphogenetic Protein 4 (BMP4), activin A (AA), and KY-02111 and XAV-939. Cells were replated on day 17 and harvested on approximately day 30.

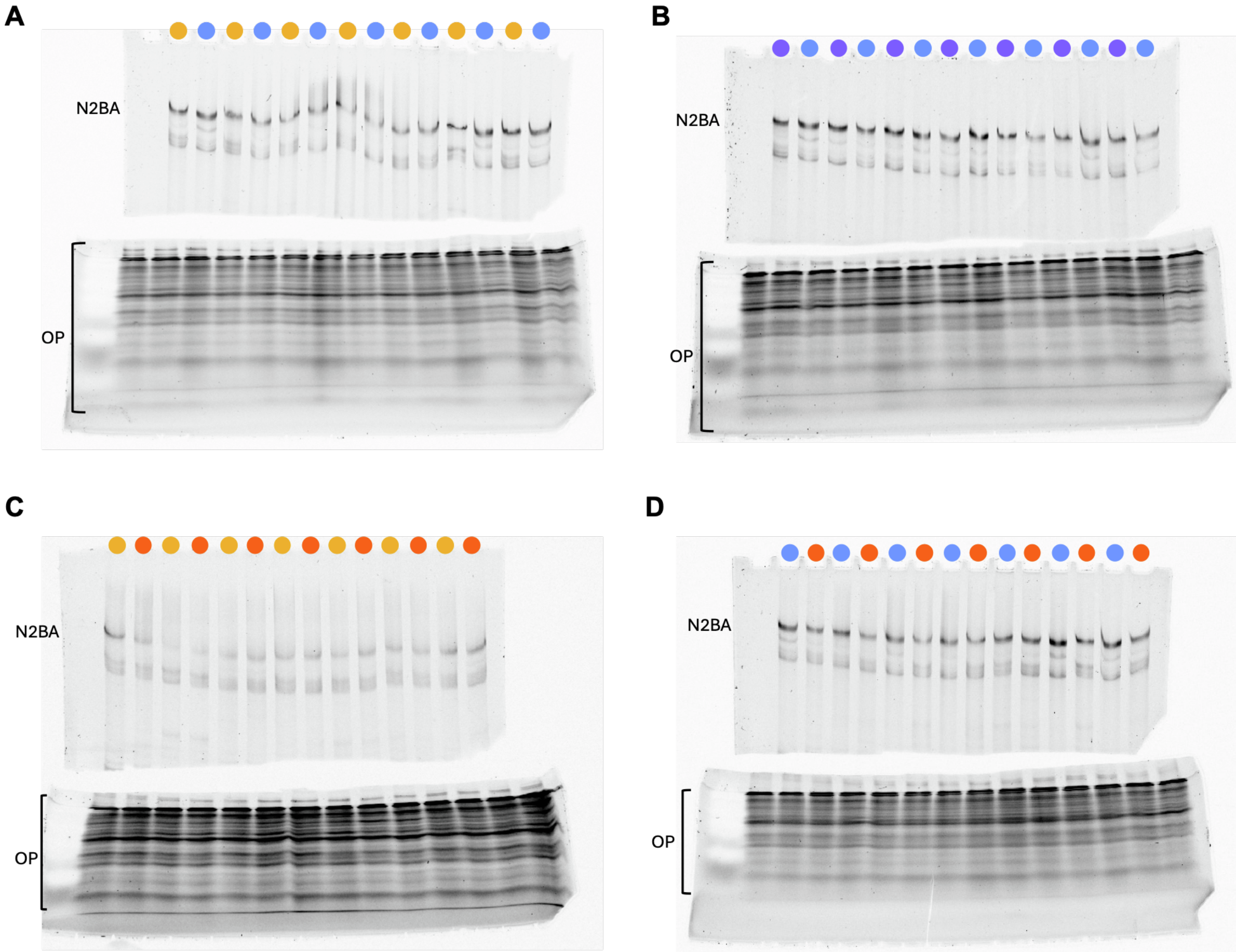

Figure S8: Uncropped versions of all gels shown in results.

Correspond to Figure 4D **(A)**, Figure 5A **(B)**, Figure 5B **(C)**, and Figure 5C **(D)**. Genotypes are colour coded – WT (blue), WT-ΔuORF (purple), *TTN*$^{+/-}$ (yellow), and *TTN*$^{+/-}$-ΔuORF (orange).
